# Supplementary figures and images for: Hyperactive PLCG1 induces cell-autonomous and bystander T cell activation and drug resistance
Source: EMBO Rep. 2025 Aug 12;26(18):4563–86. doi: 10.1038/s44319-025-00546-x (PMC12457681; doi:10.1038/s44319-025-00546-x)

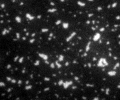

Supplement: Supplementary file 4 — Source data Fig. 1 [file 44319_2025_546_MOESM4_ESM.zip › Figure 1/Fig1C-TIRF image D1165H.tif]

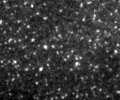

Supplement: Supplementary file 4 — Source data Fig. 1 [file 44319_2025_546_MOESM4_ESM.zip › Figure 1/Fig1C-TIRF image R48W.tif]

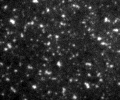

Supplement: Supplementary file 4 — Source data Fig. 1 [file 44319_2025_546_MOESM4_ESM.zip › Figure 1/Fig1C-TIRF image S345F.tif]

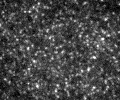

Supplement: Supplementary file 4 — Source data Fig. 1 [file 44319_2025_546_MOESM4_ESM.zip › Figure 1/Fig1C-TIRF image WT.tif]

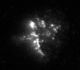

Supplement: Supplementary file 5 — Source data Fig. 2 [file 44319_2025_546_MOESM5_ESM.zip › Figure 2/Fig2B-TIRF image D1165H.tif]

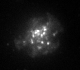

Supplement: Supplementary file 5 — Source data Fig. 2 [file 44319_2025_546_MOESM5_ESM.zip › Figure 2/Fig2B-TIRF image R48W.tif]

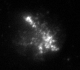

Supplement: Supplementary file 5 — Source data Fig. 2 [file 44319_2025_546_MOESM5_ESM.zip › Figure 2/Fig2B-TIRF image S345F.tif]

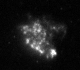

Supplement: Supplementary file 5 — Source data Fig. 2 [file 44319_2025_546_MOESM5_ESM.zip › Figure 2/Fig2B-TIRF image WT.tif]

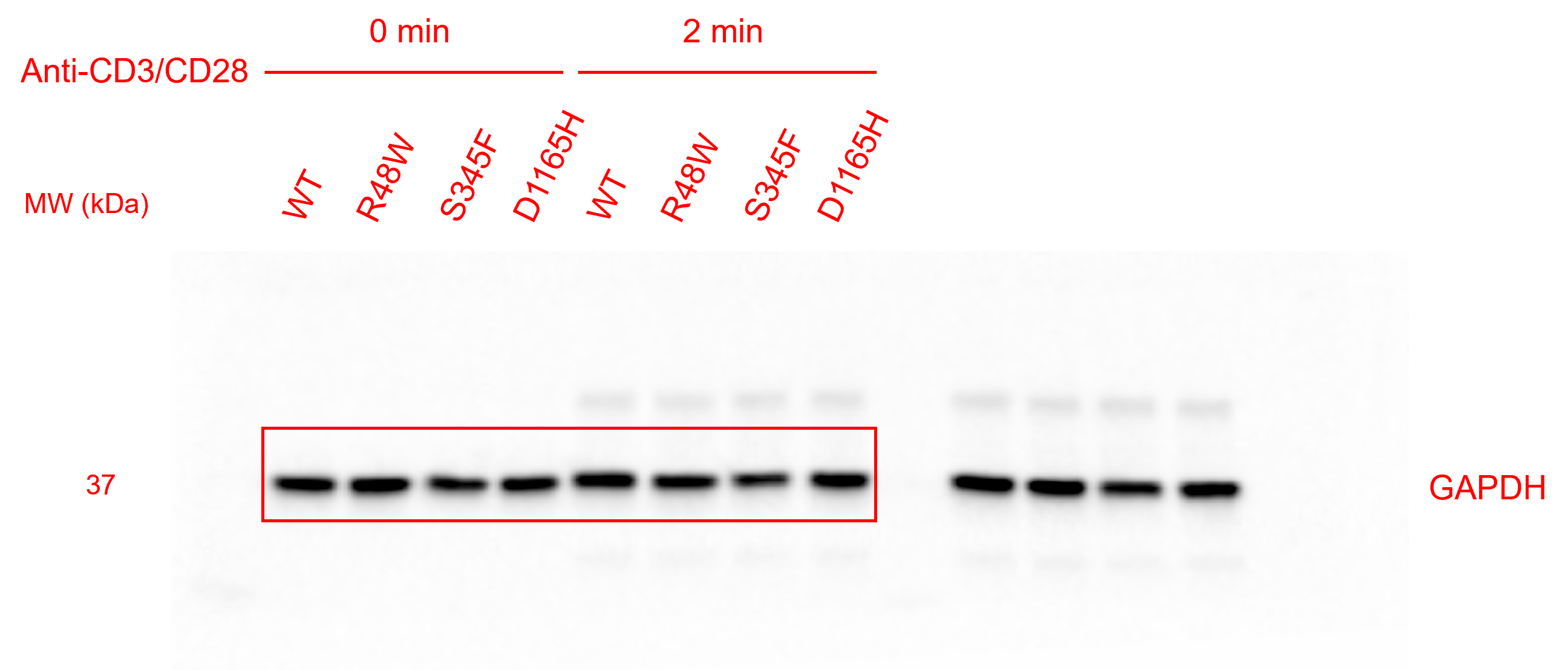

Supplement: Supplementary file 5 — Source data Fig. 2 [file 44319_2025_546_MOESM5_ESM.zip › Figure 2/Fig2D-Western GAPDH.png]

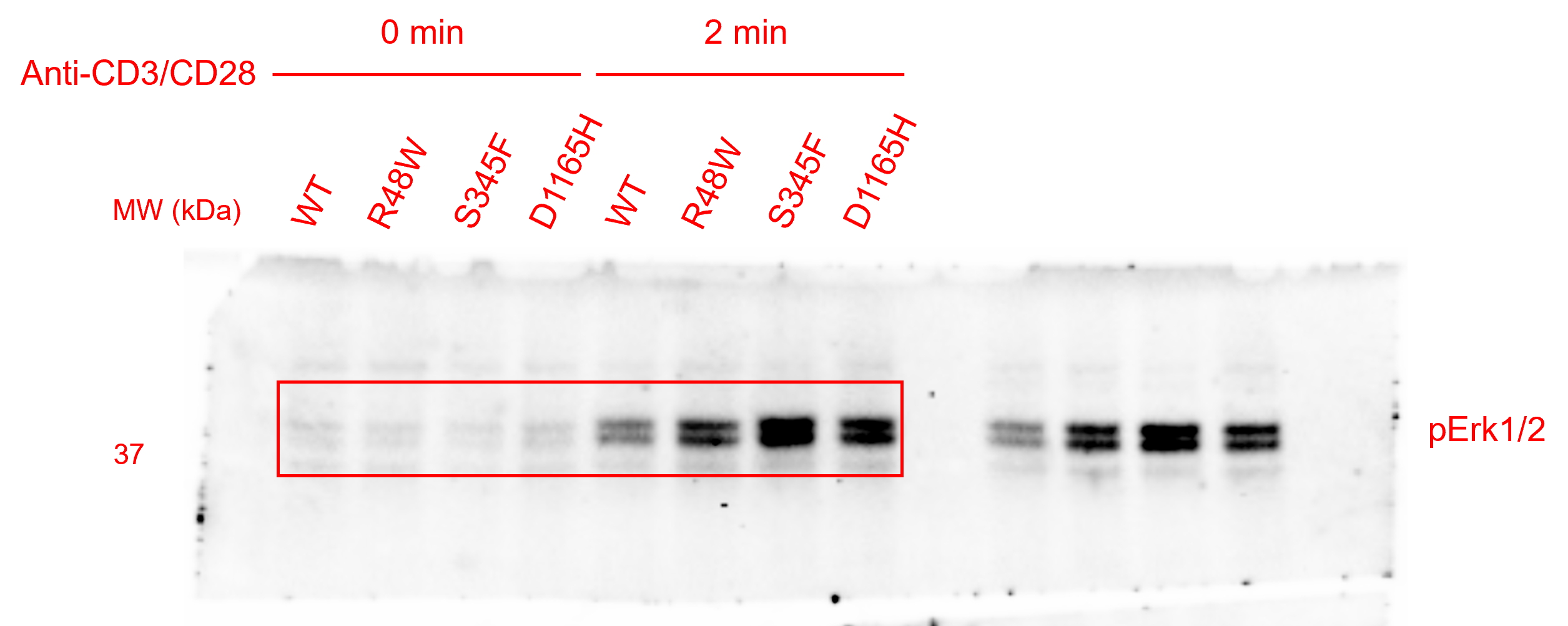

Supplement: Supplementary file 5 — Source data Fig. 2 [file 44319_2025_546_MOESM5_ESM.zip › Figure 2/Fig2D-Western pERK.png]

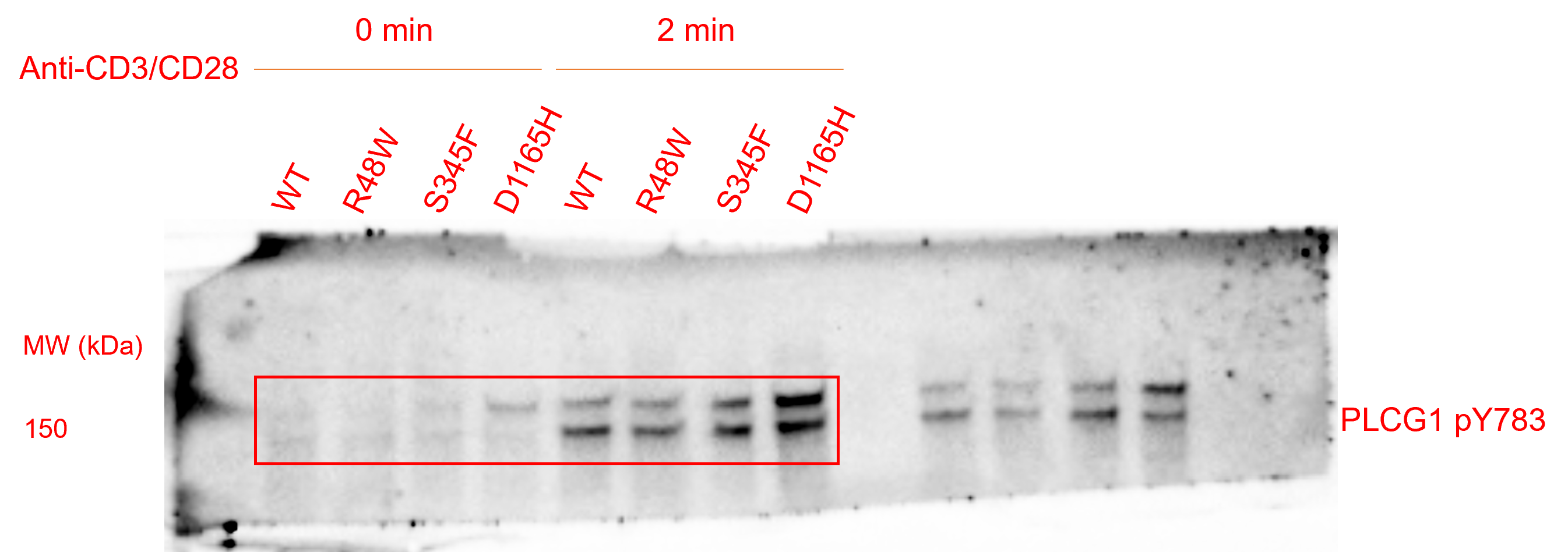

Supplement: Supplementary file 5 — Source data Fig. 2 [file 44319_2025_546_MOESM5_ESM.zip › Figure 2/Fig2D-Western PLCG1 pY783.png]

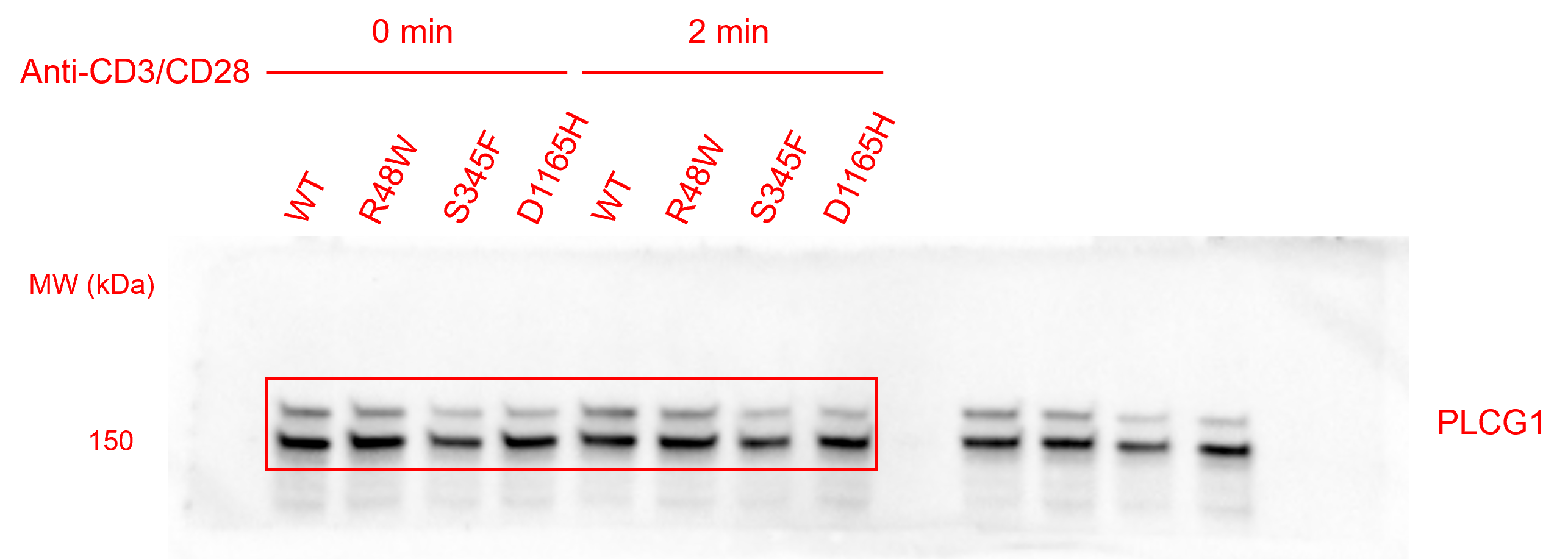

Supplement: Supplementary file 5 — Source data Fig. 2 [file 44319_2025_546_MOESM5_ESM.zip › Figure 2/Fig2D-Western PLCG1.png]

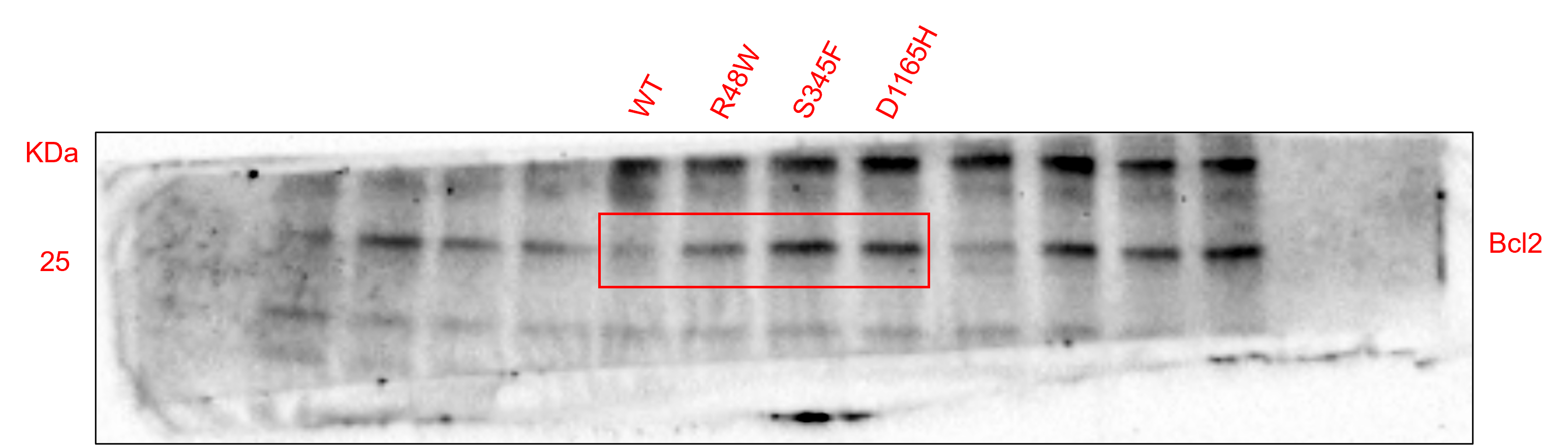

Supplement: Supplementary file 6 — Source data Fig. 3 [file 44319_2025_546_MOESM6_ESM.zip › Figure 3/Fig3A-Western Bcl2.png]

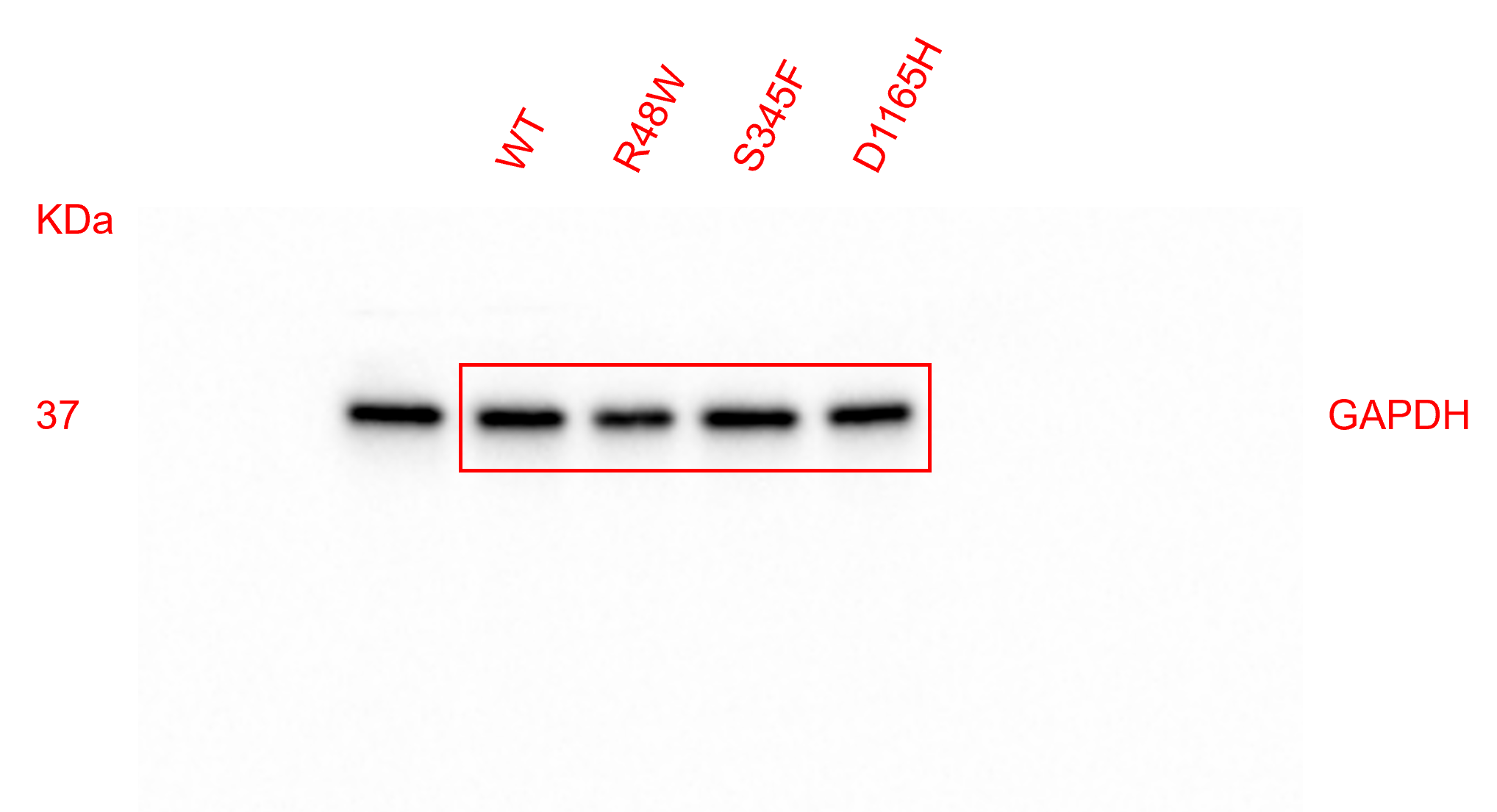

Supplement: Supplementary file 6 — Source data Fig. 3 [file 44319_2025_546_MOESM6_ESM.zip › Figure 3/Fig3A-Western GAPDH.png]

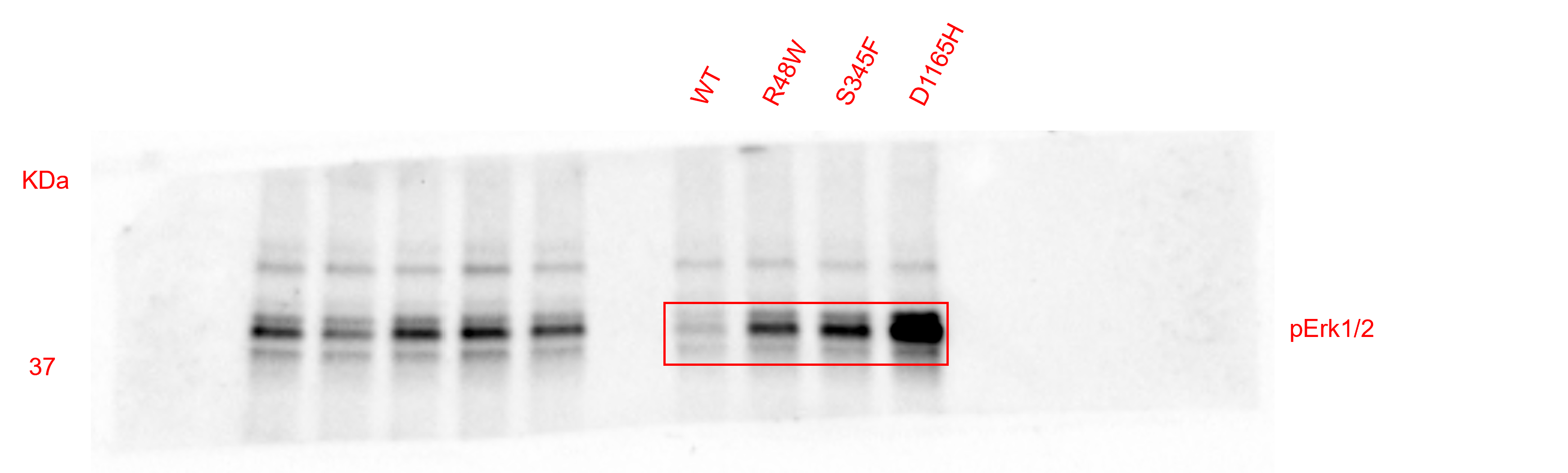

Supplement: Supplementary file 6 — Source data Fig. 3 [file 44319_2025_546_MOESM6_ESM.zip › Figure 3/Fig3A-Western pERK.png]

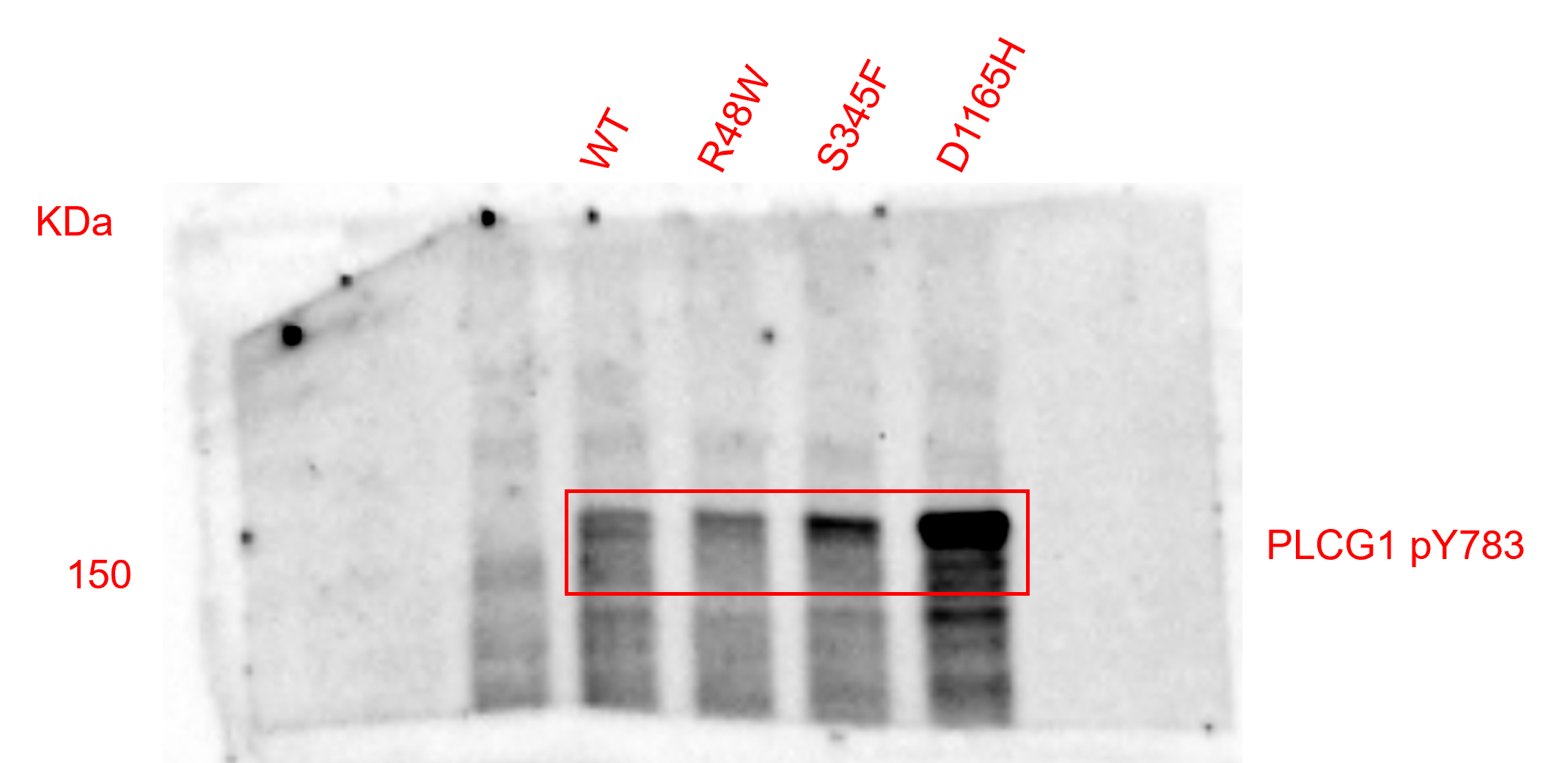

Supplement: Supplementary file 6 — Source data Fig. 3 [file 44319_2025_546_MOESM6_ESM.zip › Figure 3/Fig3A-Western PLCG1 pY783.png]

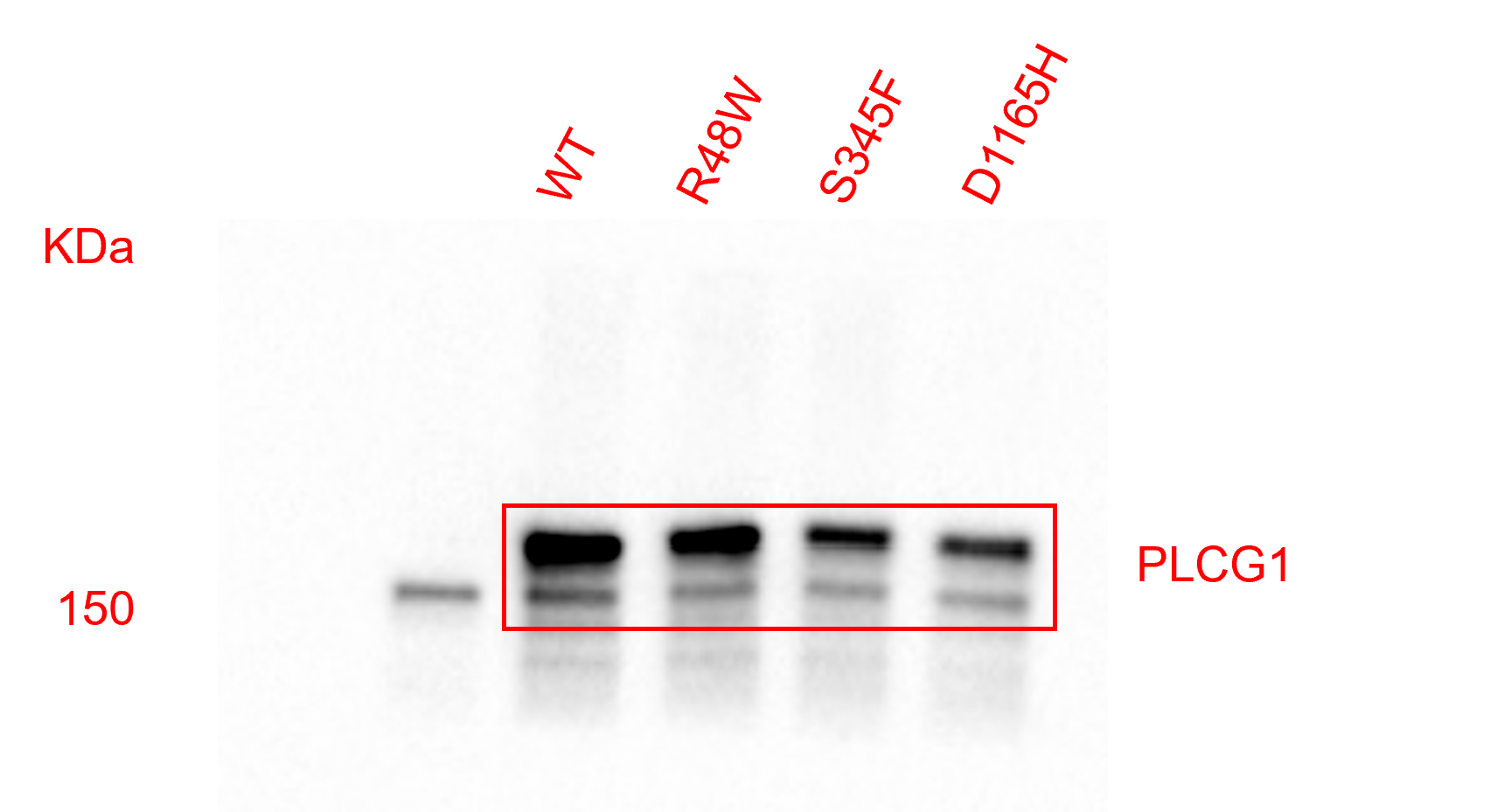

Supplement: Supplementary file 6 — Source data Fig. 3 [file 44319_2025_546_MOESM6_ESM.zip › Figure 3/Fig3A-Western PLCG1.png]

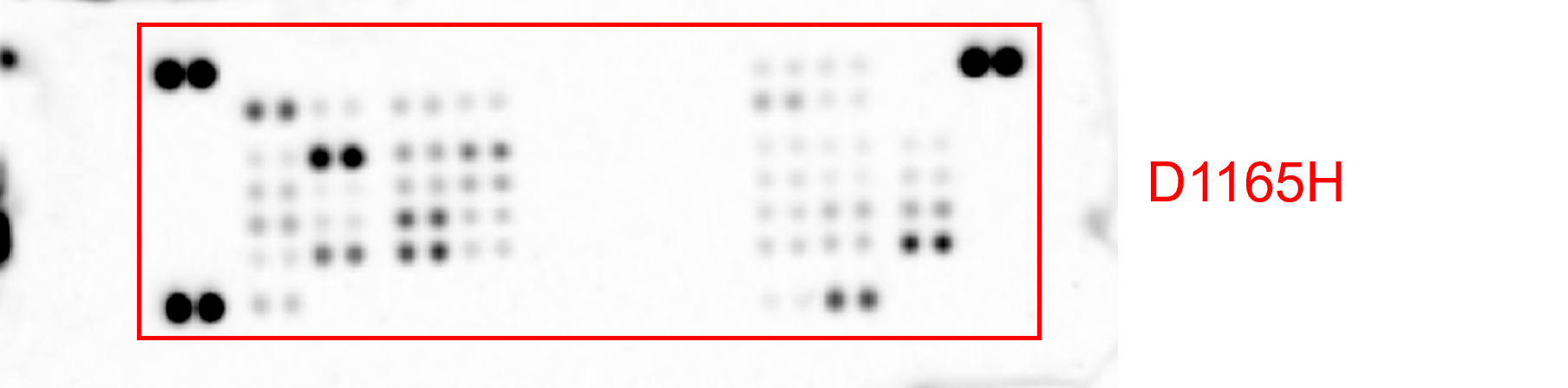

Supplement: Supplementary file 6 — Source data Fig. 3 [file 44319_2025_546_MOESM6_ESM.zip › Figure 3/Fig3B-Human phospho-kinase array D1165H.png]

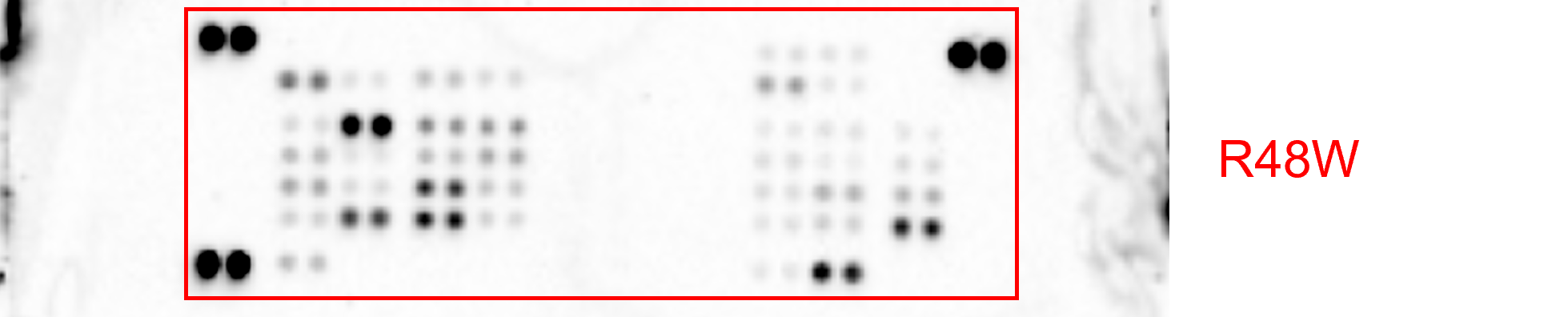

Supplement: Supplementary file 6 — Source data Fig. 3 [file 44319_2025_546_MOESM6_ESM.zip › Figure 3/Fig3B-Human phospho-kinase array R48W.png]

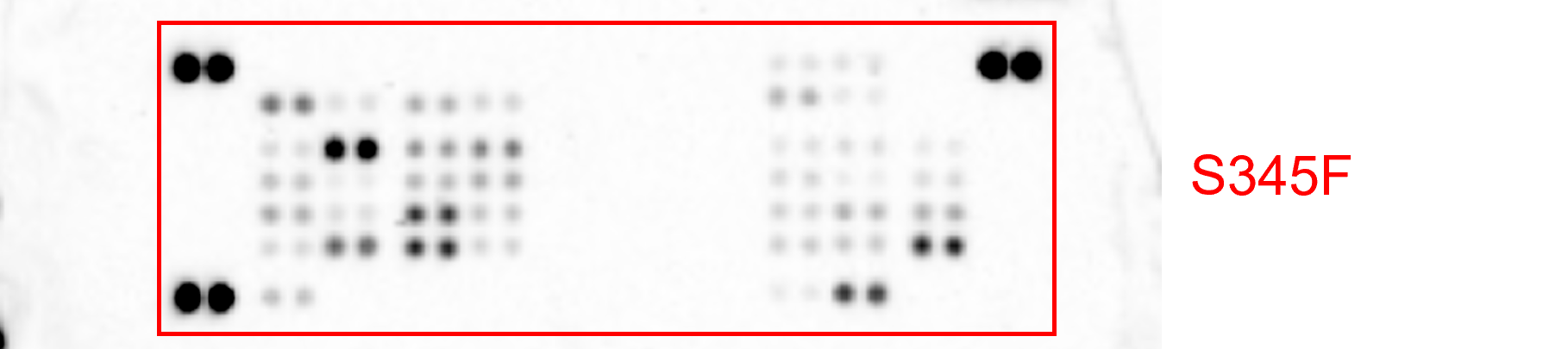

Supplement: Supplementary file 6 — Source data Fig. 3 [file 44319_2025_546_MOESM6_ESM.zip › Figure 3/Fig3B-Human phospho-kinase array S345F.png]

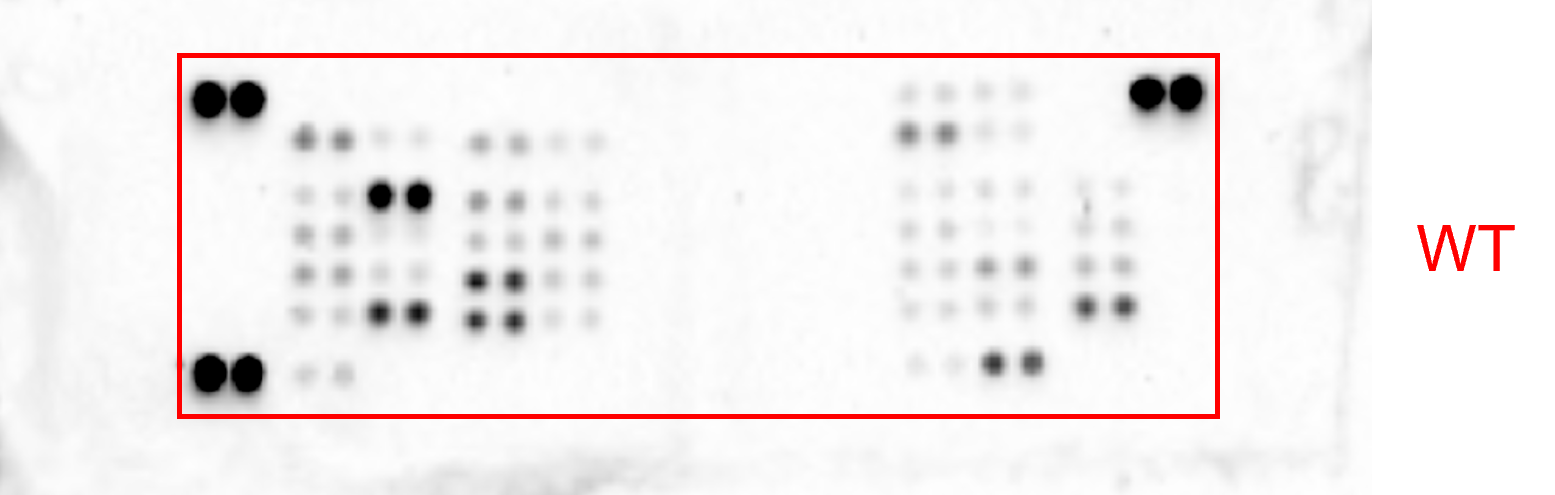

Supplement: Supplementary file 6 — Source data Fig. 3 [file 44319_2025_546_MOESM6_ESM.zip › Figure 3/Fig3B-Human phospho-kinase array WT.png]

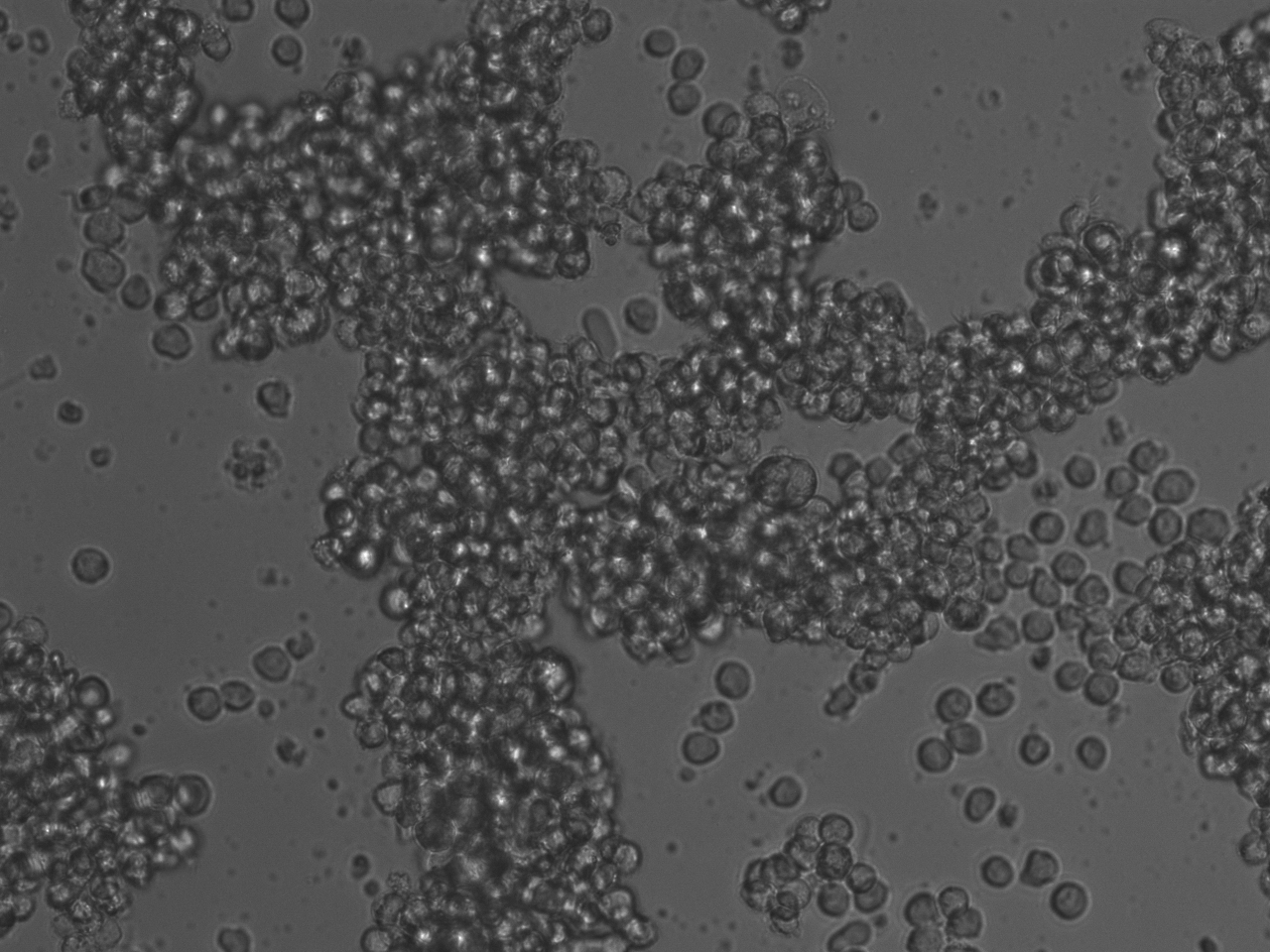

Supplement: Supplementary file 7 — Source data Fig. 4 [file 44319_2025_546_MOESM7_ESM.zip › Figure 4/Fig4A-Imaging D1165H.tif]

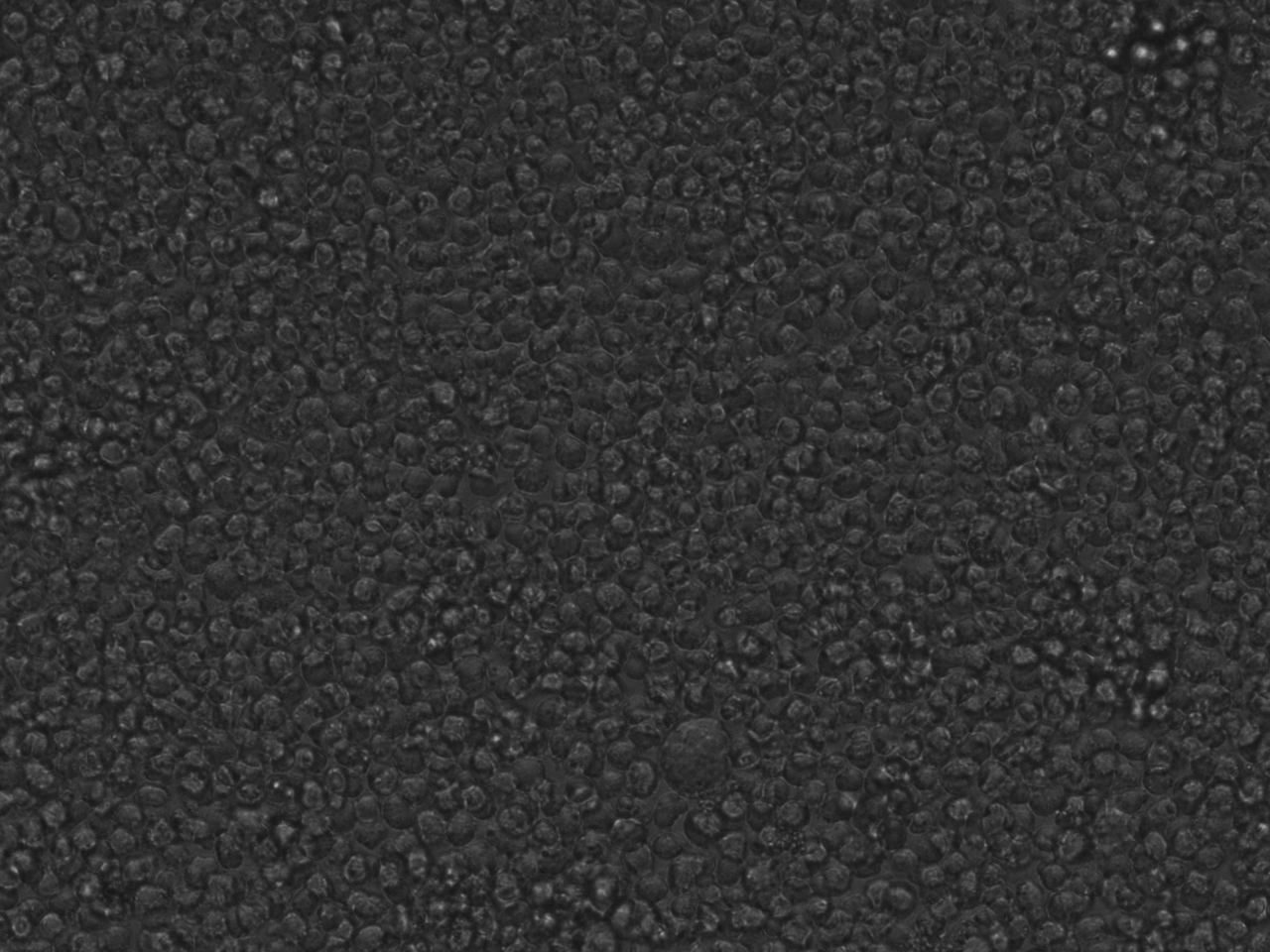

Supplement: Supplementary file 7 — Source data Fig. 4 [file 44319_2025_546_MOESM7_ESM.zip › Figure 4/Fig4A-Imaging Plain.tif]

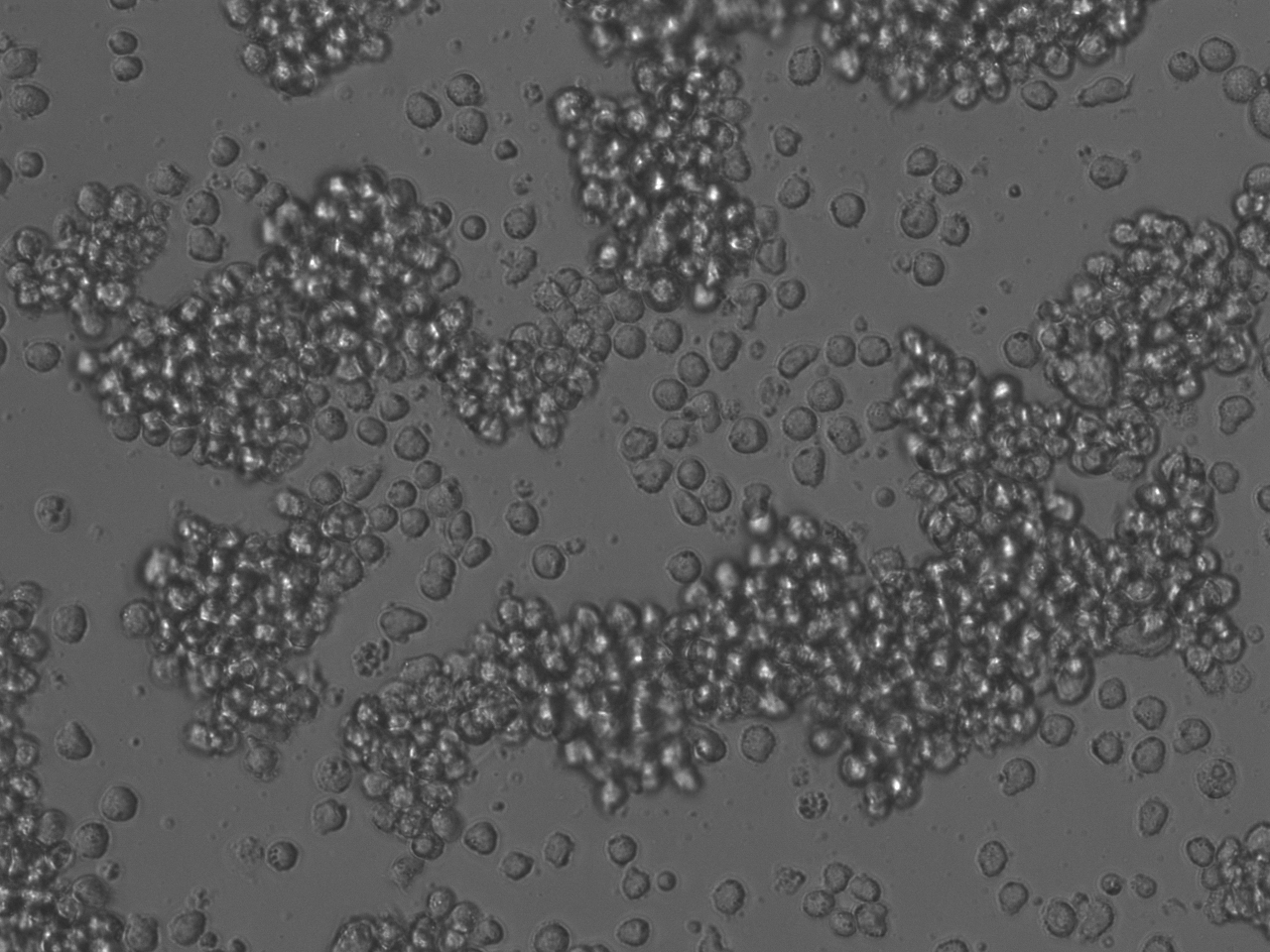

Supplement: Supplementary file 7 — Source data Fig. 4 [file 44319_2025_546_MOESM7_ESM.zip › Figure 4/Fig4A-Imaging R48W.tif]

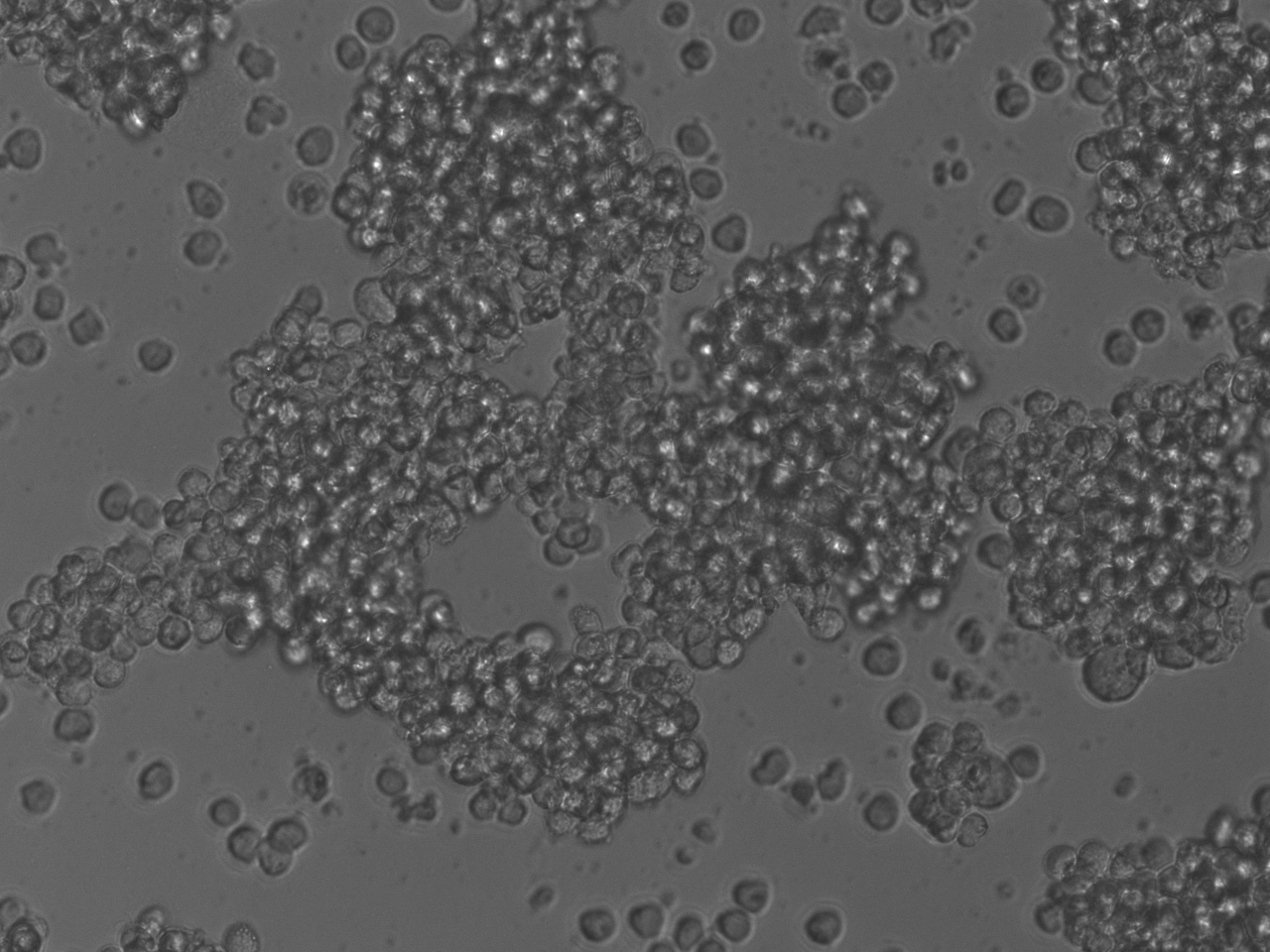

Supplement: Supplementary file 7 — Source data Fig. 4 [file 44319_2025_546_MOESM7_ESM.zip › Figure 4/Fig4A-Imaging S345F.tif]

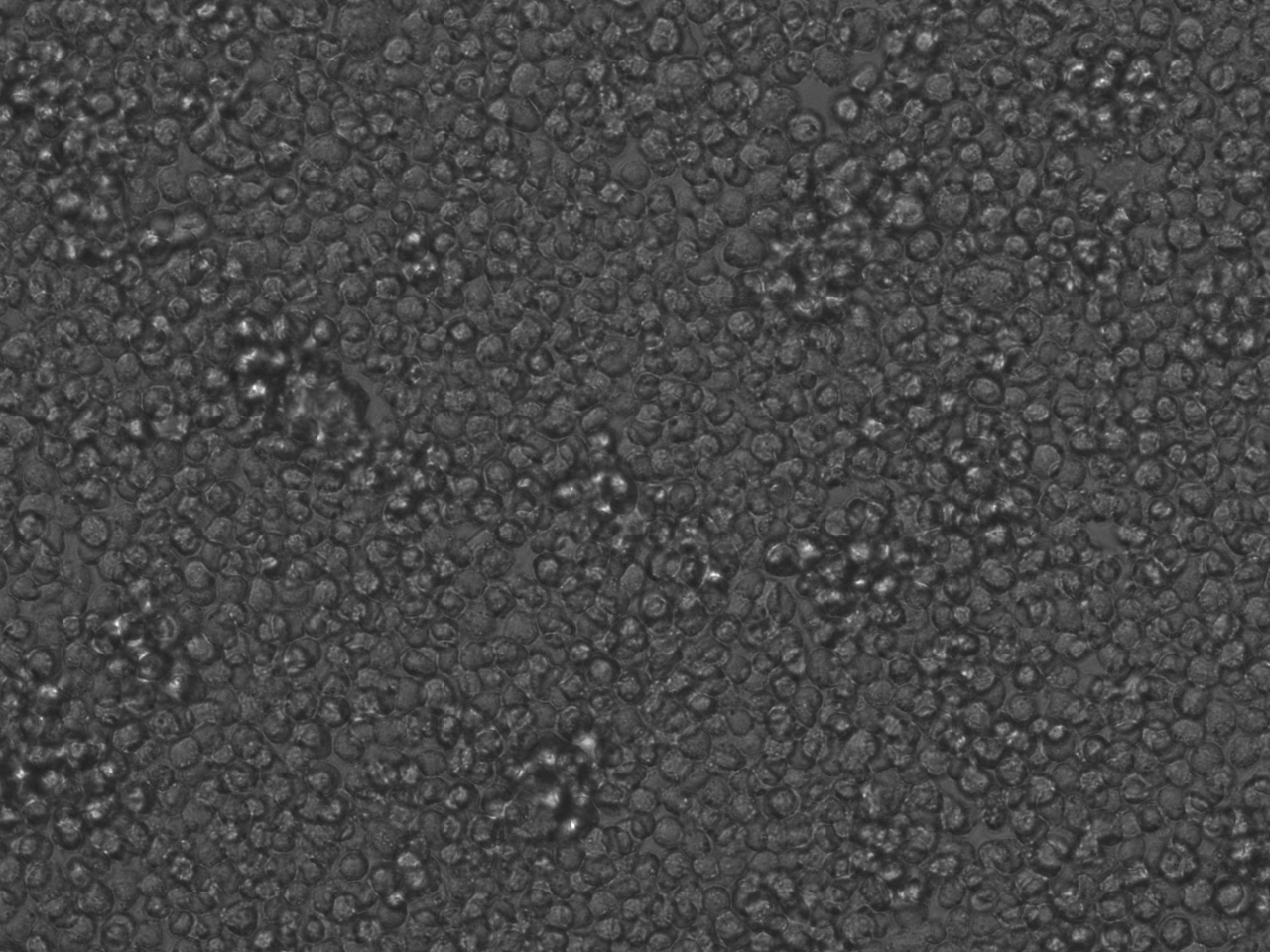

Supplement: Supplementary file 7 — Source data Fig. 4 [file 44319_2025_546_MOESM7_ESM.zip › Figure 4/Fig4A-Imaging WT.tif]

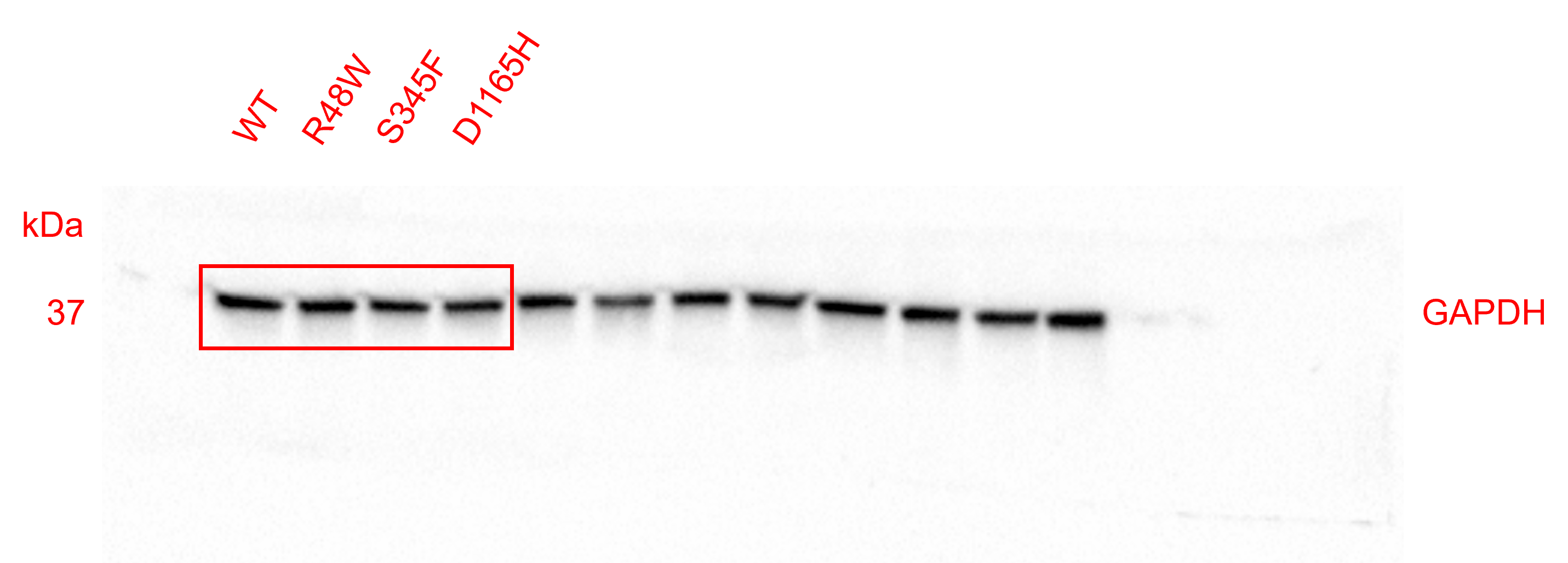

Supplement: Supplementary file 7 — Source data Fig. 4 [file 44319_2025_546_MOESM7_ESM.zip › Figure 4/Fig4E-Western GAPDH.png]

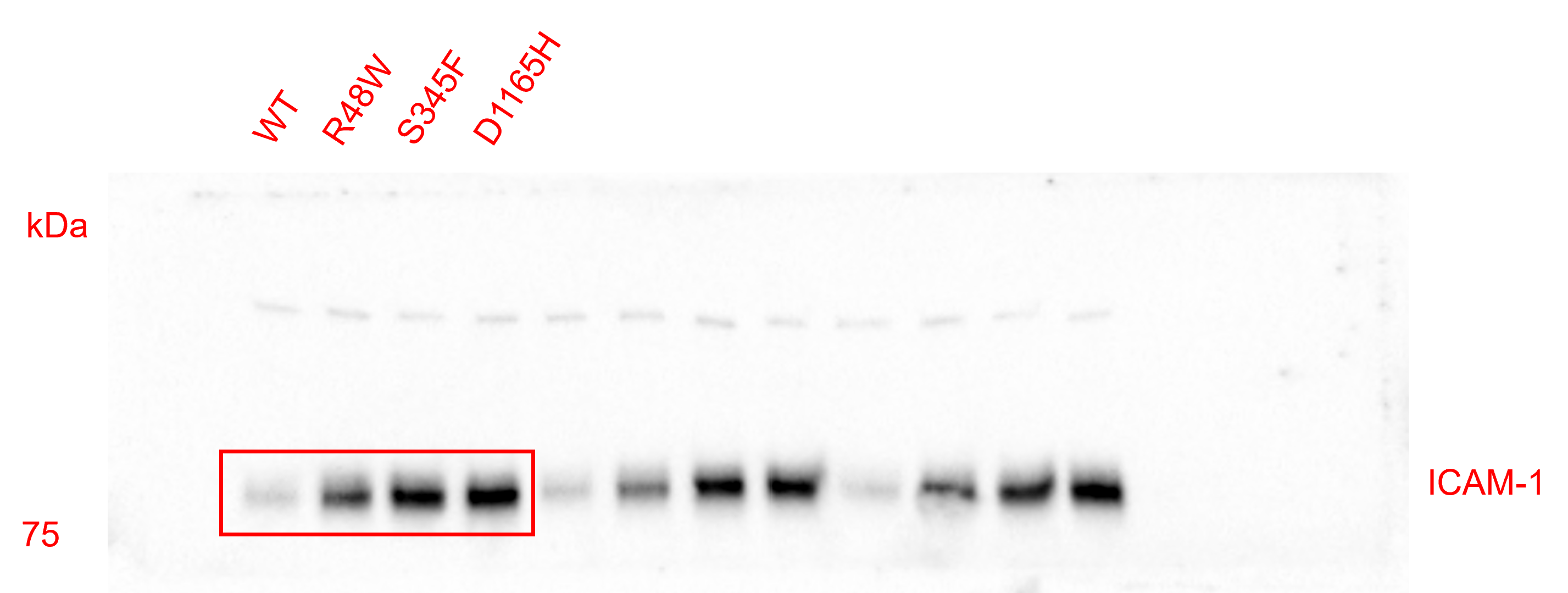

Supplement: Supplementary file 7 — Source data Fig. 4 [file 44319_2025_546_MOESM7_ESM.zip › Figure 4/Fig4E-Western IACM-1.png]

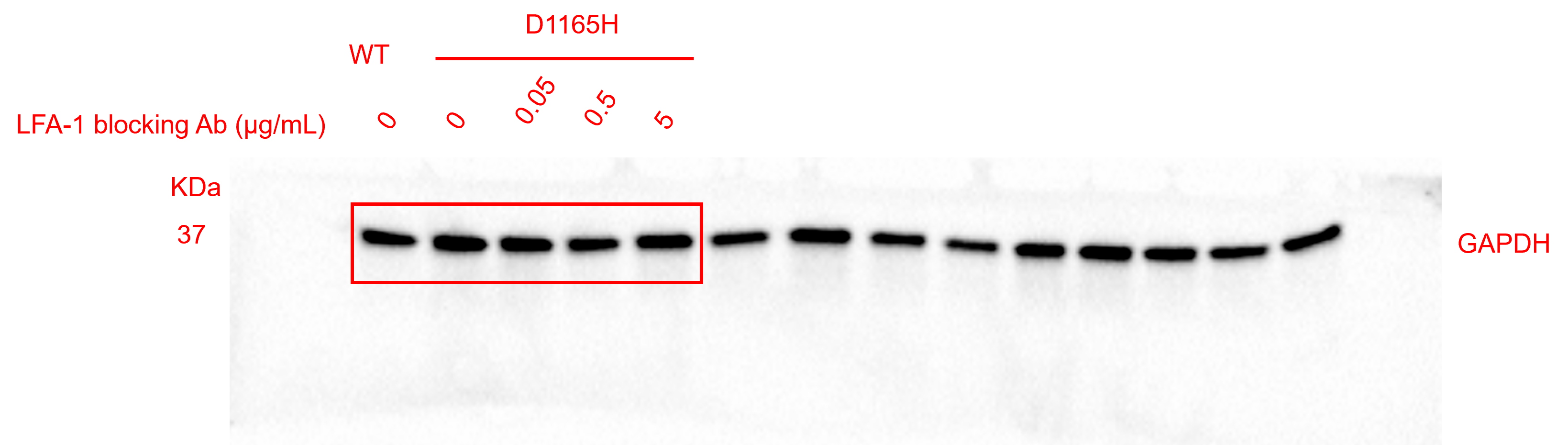

Supplement: Supplementary file 7 — Source data Fig. 4 [file 44319_2025_546_MOESM7_ESM.zip › Figure 4/Fig4G-Western GAPDH.png]

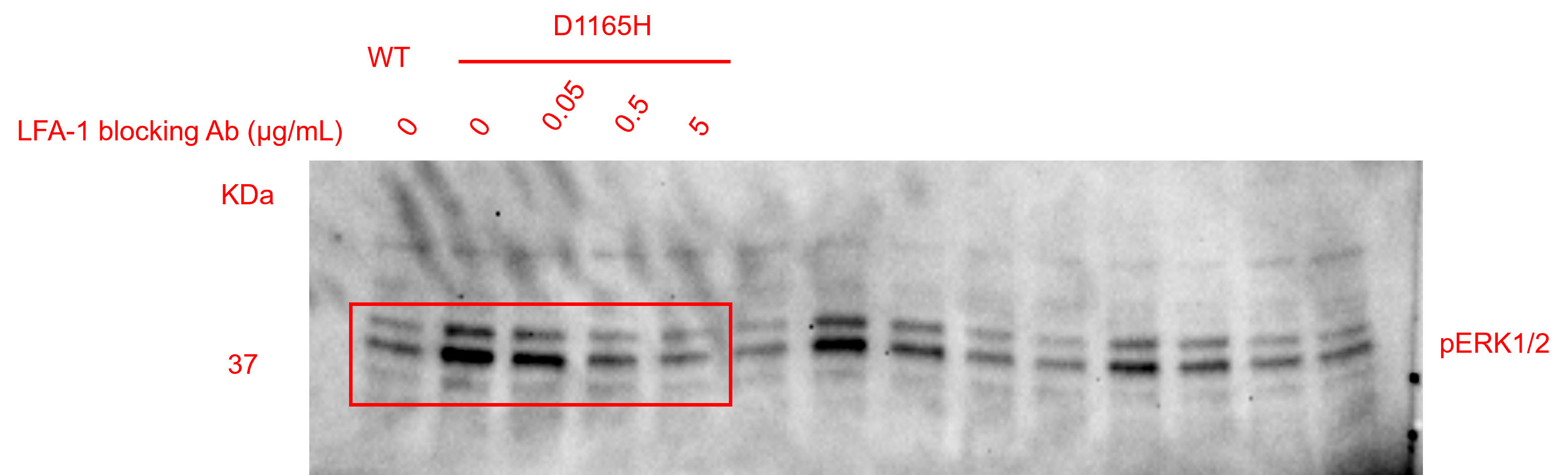

Supplement: Supplementary file 7 — Source data Fig. 4 [file 44319_2025_546_MOESM7_ESM.zip › Figure 4/Fig4G-Western pERK.png]

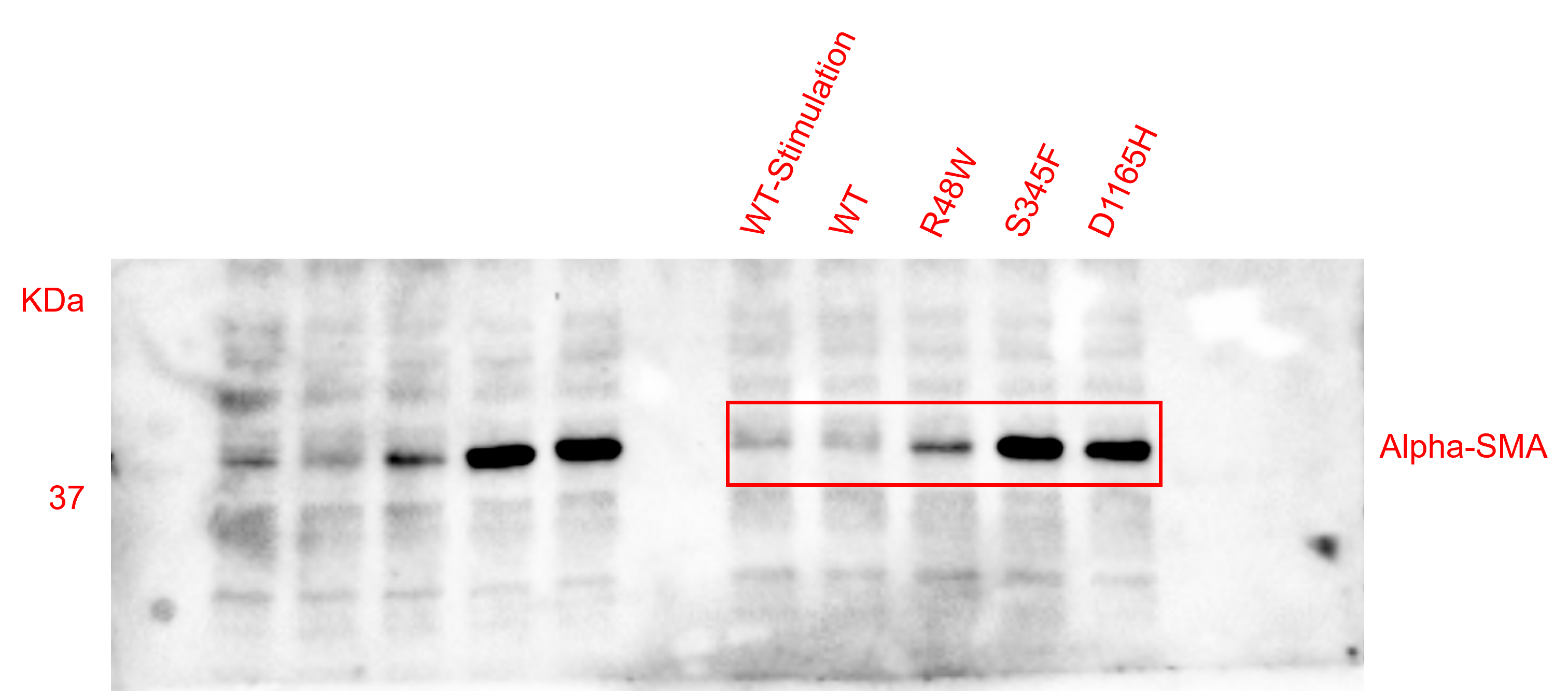

Supplement: Supplementary file 9 — Source data Fig. 6 [file 44319_2025_546_MOESM9_ESM.zip › Figure 6/Fig6D-Western Alpha-SMA.png]

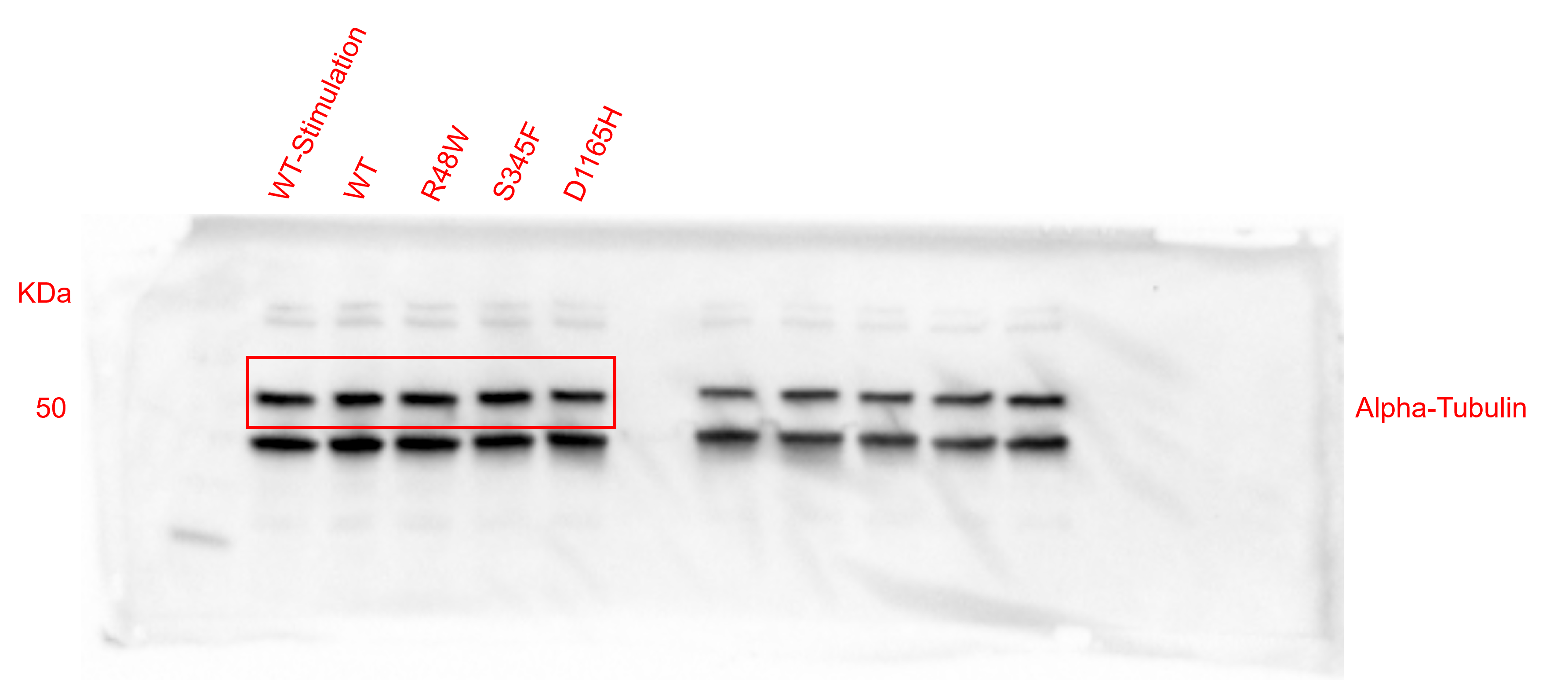

Supplement: Supplementary file 9 — Source data Fig. 6 [file 44319_2025_546_MOESM9_ESM.zip › Figure 6/Fig6D-Western Alpha-Tubulin.png]

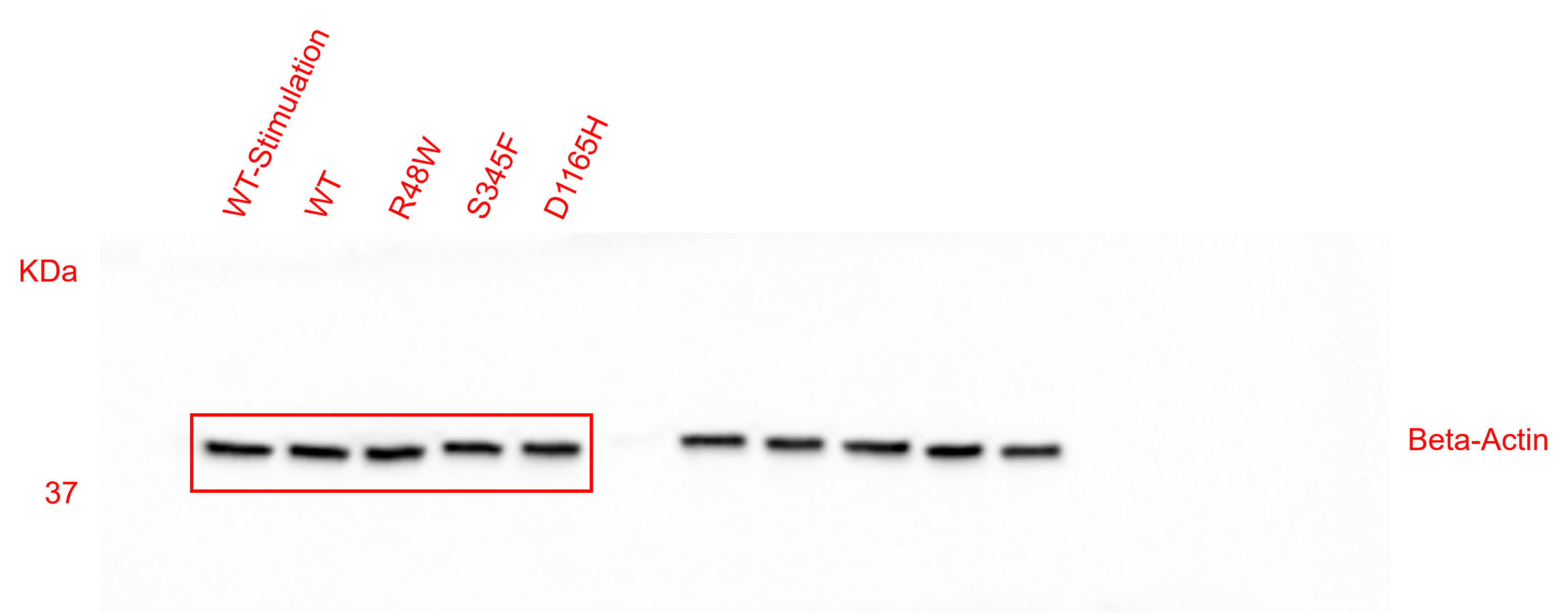

Supplement: Supplementary file 9 — Source data Fig. 6 [file 44319_2025_546_MOESM9_ESM.zip › Figure 6/Fig6D-Western Beta-Actin.png]

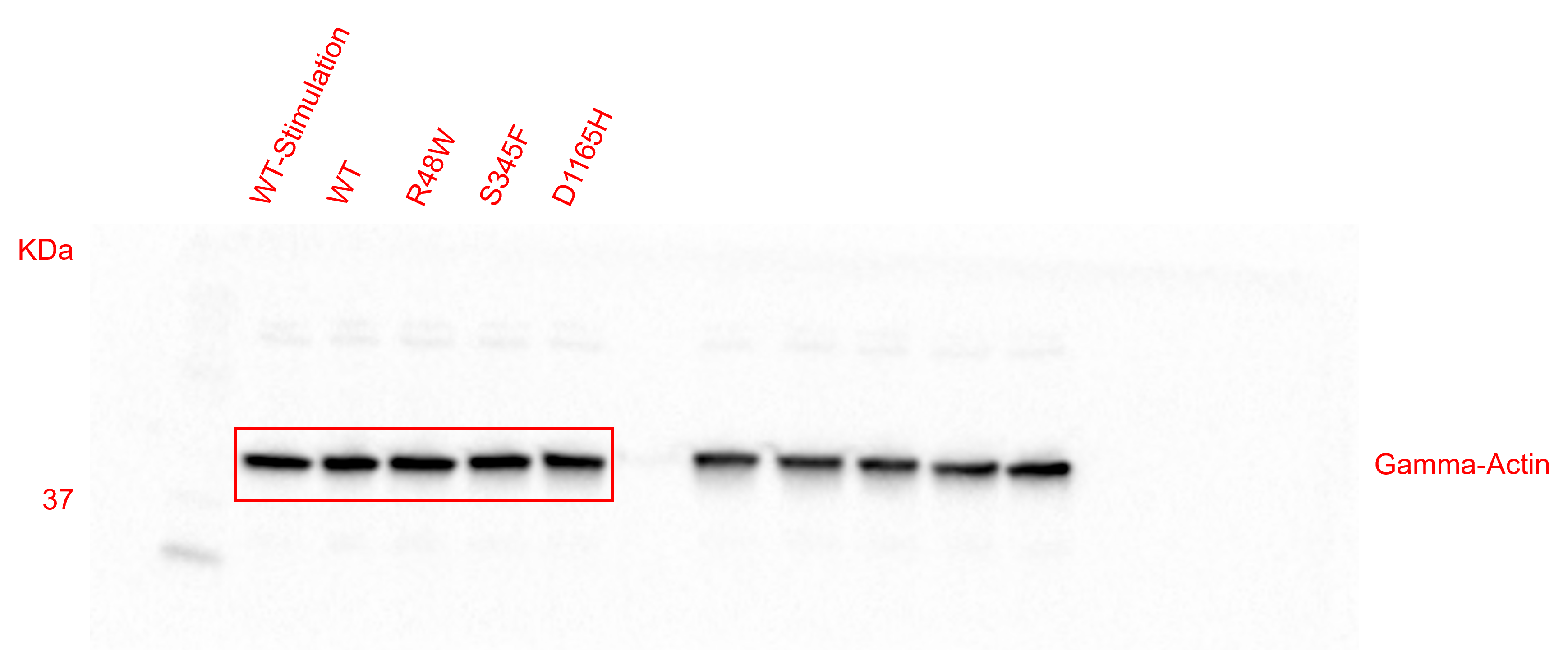

Supplement: Supplementary file 9 — Source data Fig. 6 [file 44319_2025_546_MOESM9_ESM.zip › Figure 6/Fig6D-Western Gamma-Actin.png]

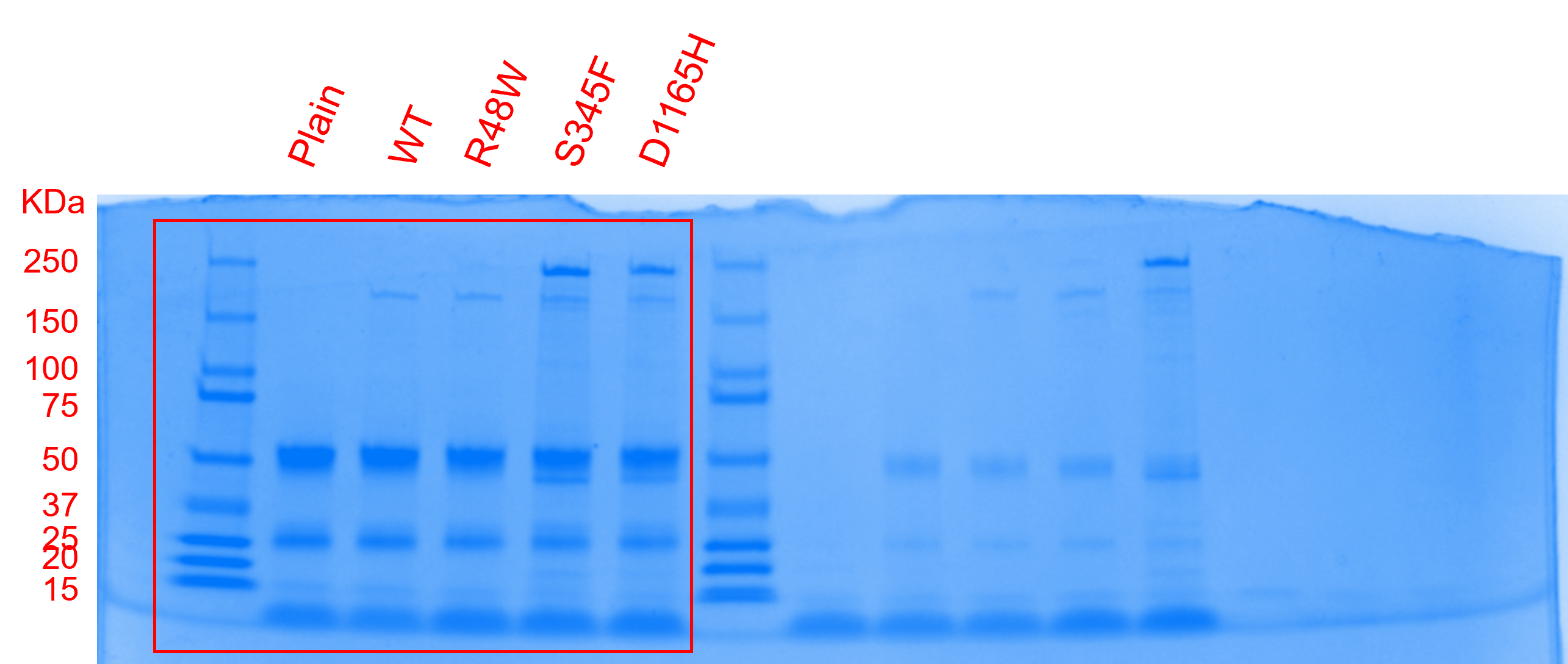

Supplement: Supplementary file 10 — Source data Fig. 7 [file 44319_2025_546_MOESM10_ESM.zip › Figure 7/Fig7A-Pull down Gel image.png]

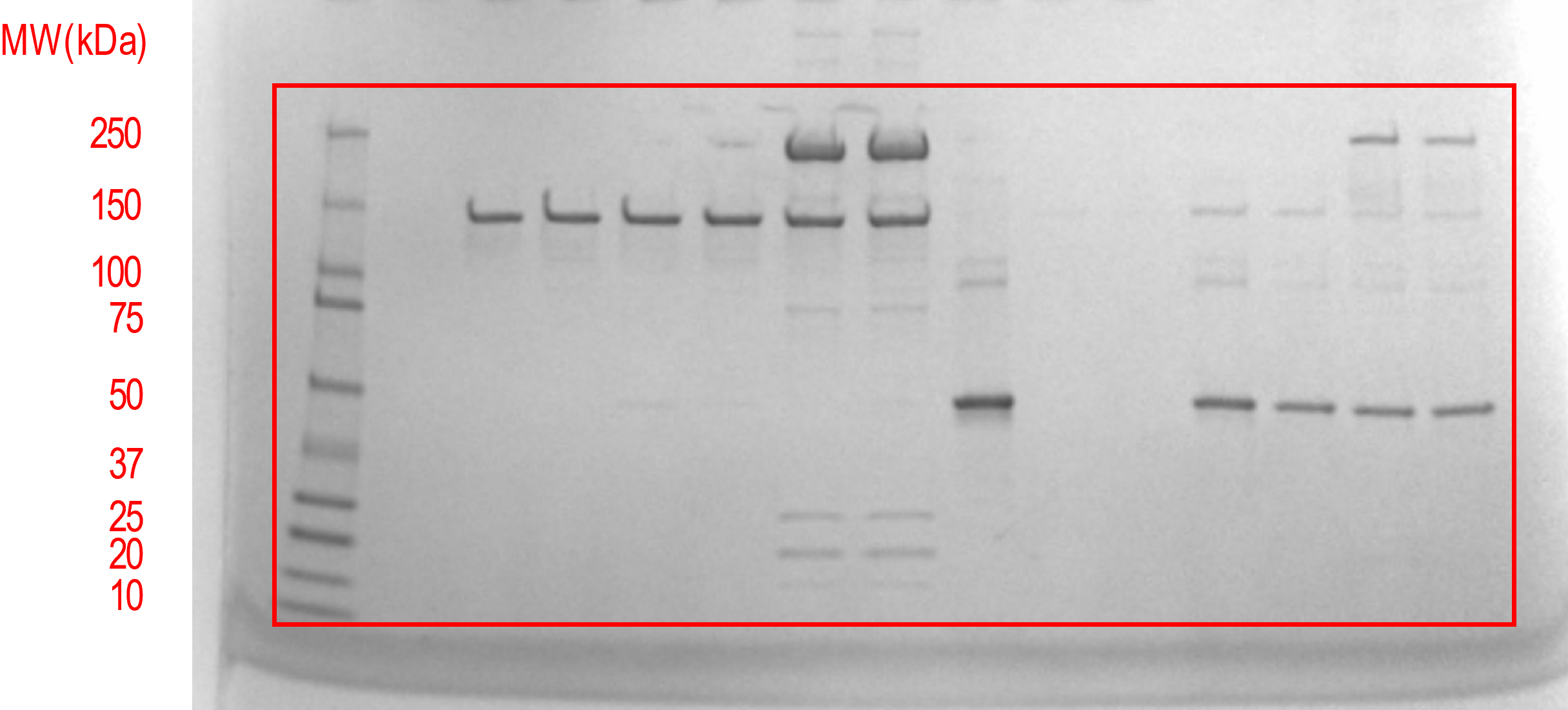

Supplement: Supplementary file 10 — Source data Fig. 7 [file 44319_2025_546_MOESM10_ESM.zip › Figure 7/Fig7B-Actin binding assay.png]

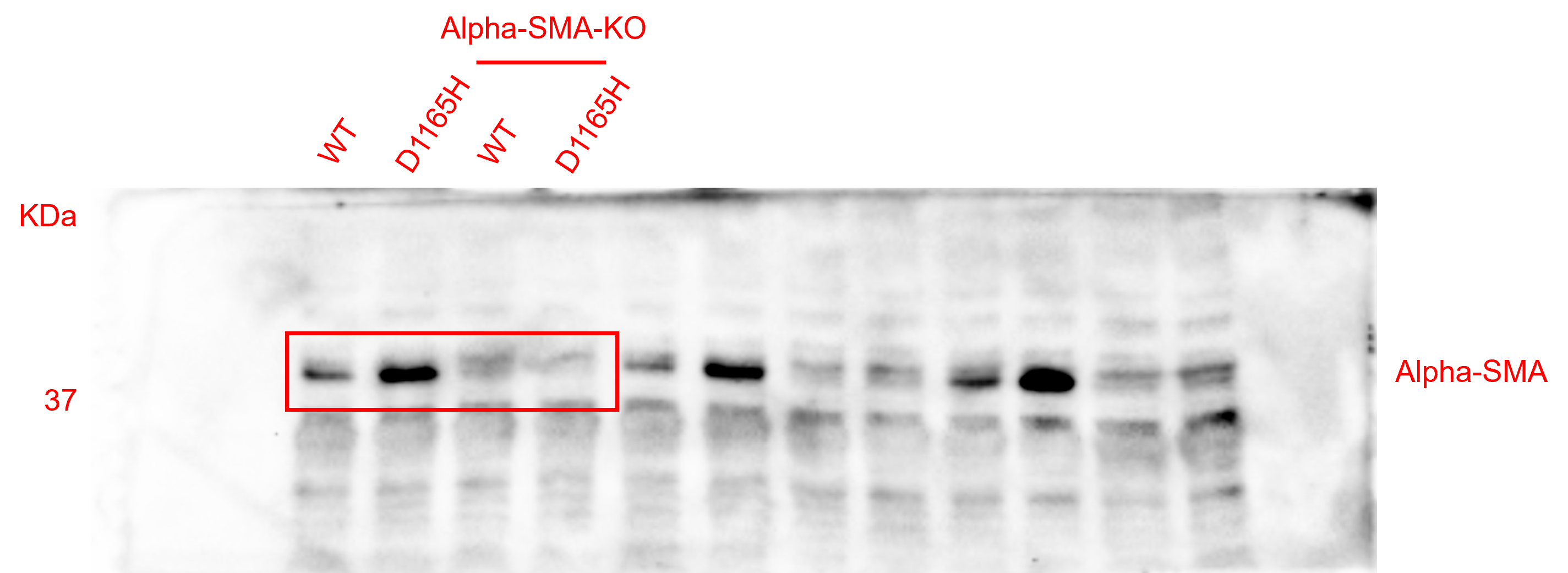

Supplement: Supplementary file 10 — Source data Fig. 7 [file 44319_2025_546_MOESM10_ESM.zip › Figure 7/Fig7D-Western Alpha-SMA.png]

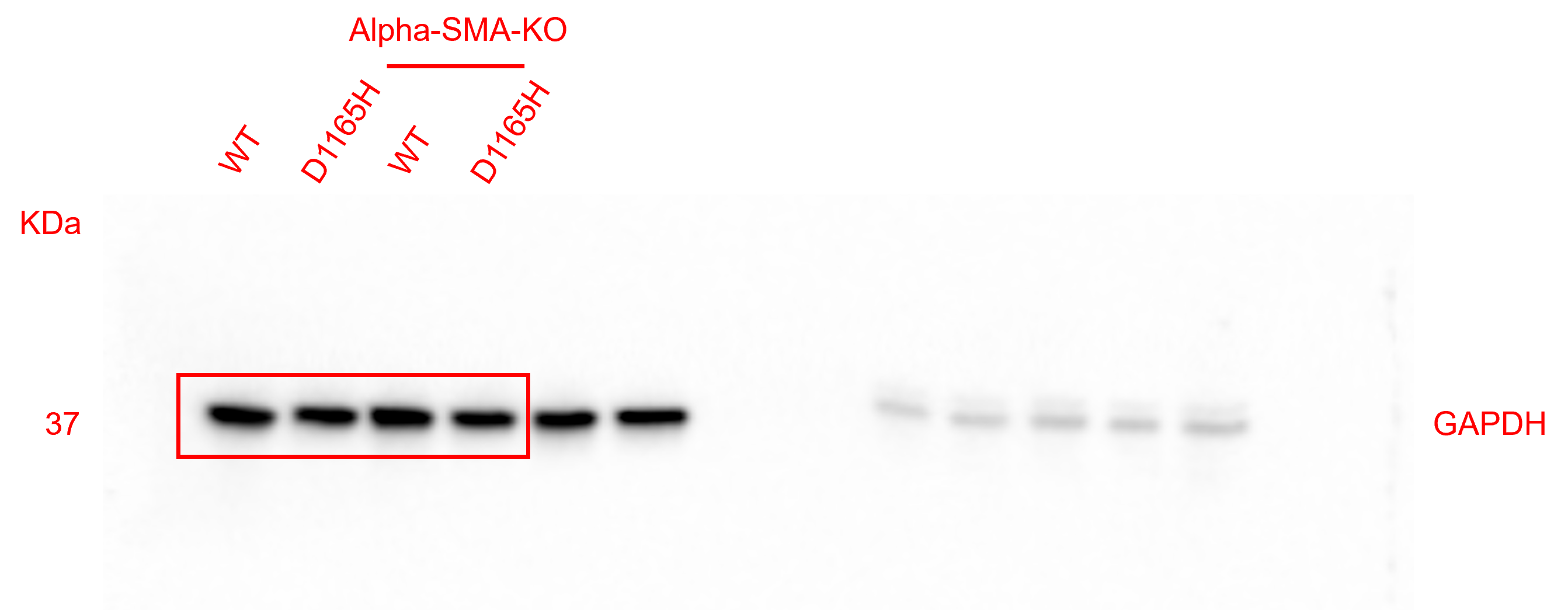

Supplement: Supplementary file 10 — Source data Fig. 7 [file 44319_2025_546_MOESM10_ESM.zip › Figure 7/Fig7D-Western GAPDH.png]

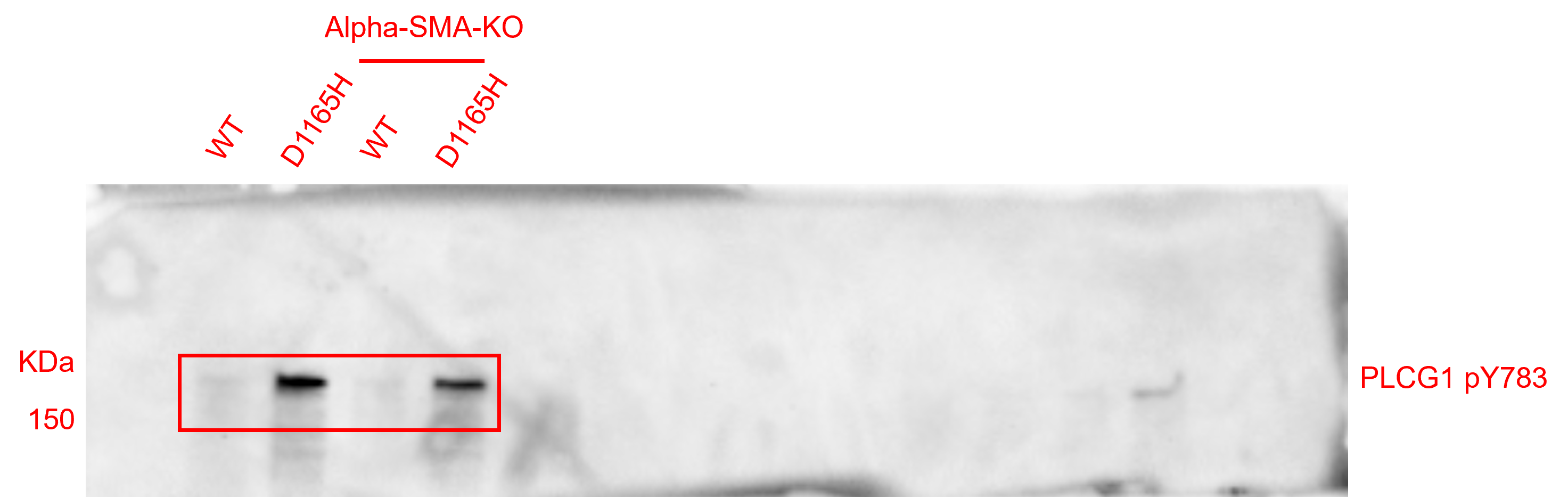

Supplement: Supplementary file 10 — Source data Fig. 7 [file 44319_2025_546_MOESM10_ESM.zip › Figure 7/Fig7D-Western PLCG1 pY783.png]

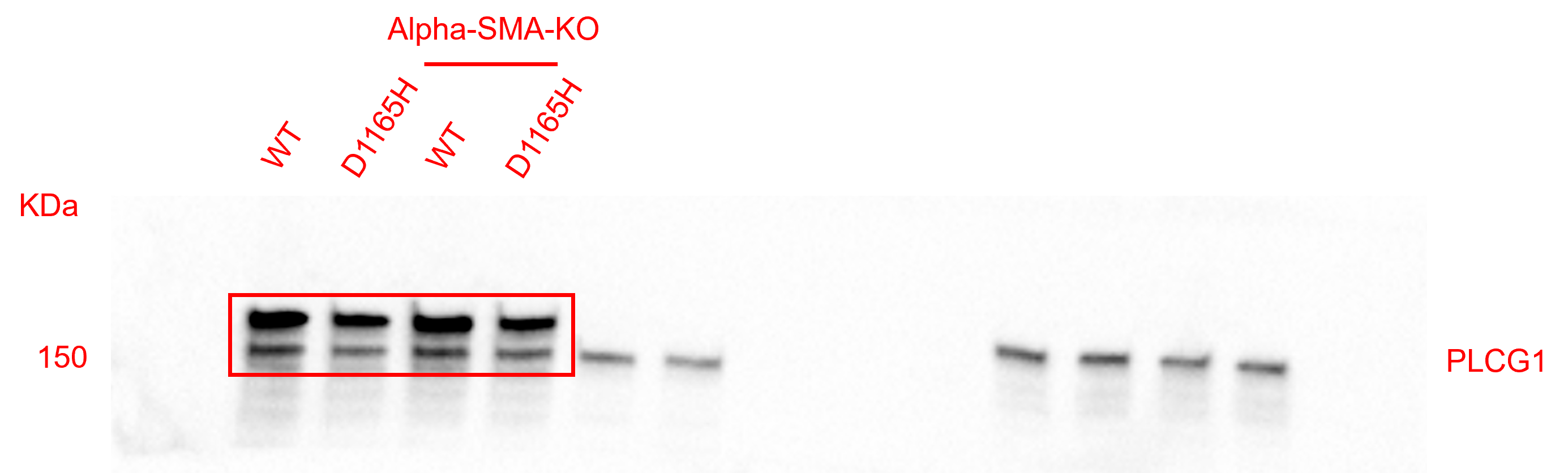

Supplement: Supplementary file 10 — Source data Fig. 7 [file 44319_2025_546_MOESM10_ESM.zip › Figure 7/Fig7D-Western PLCG1.png]

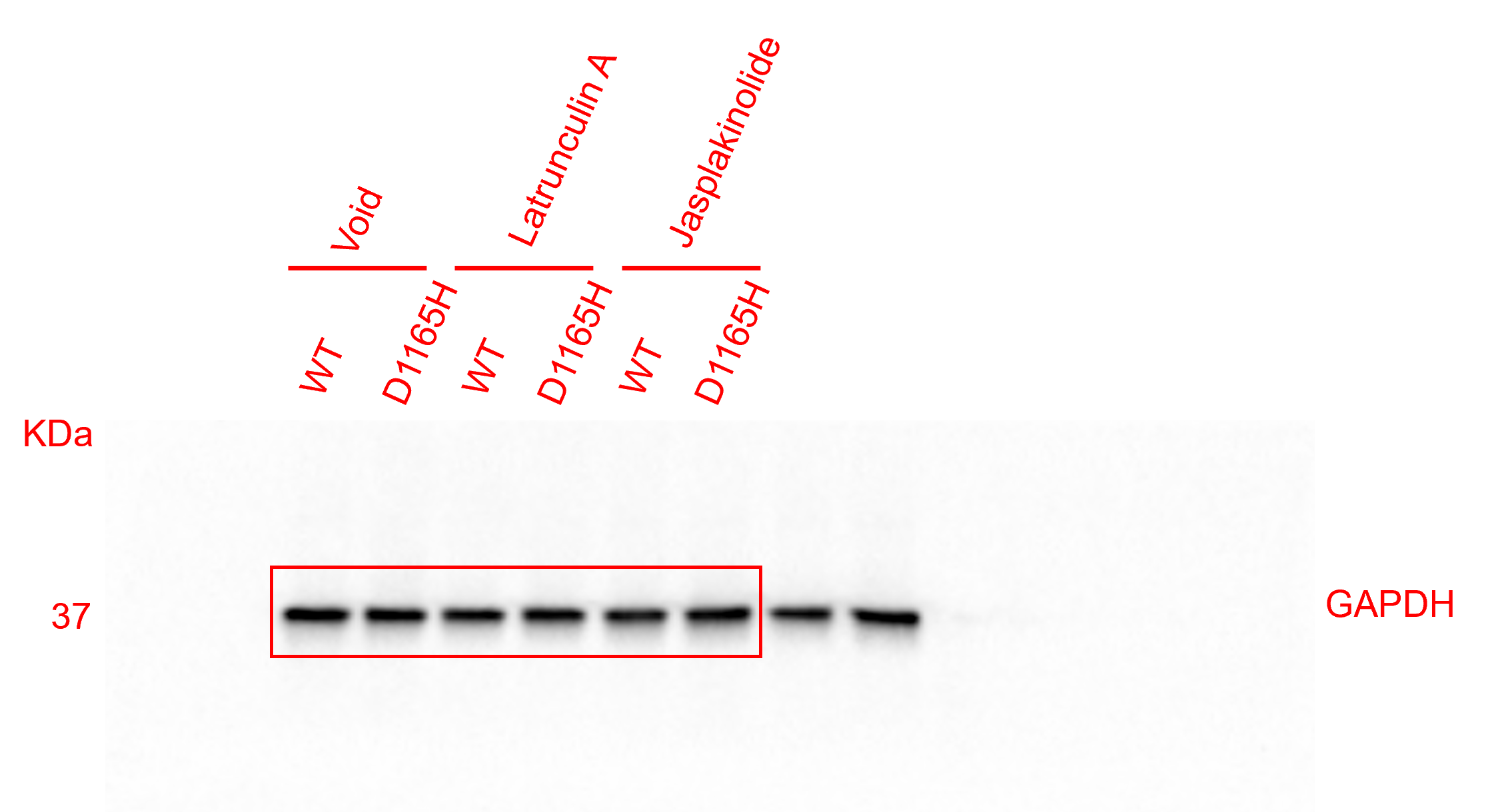

Supplement: Supplementary file 10 — Source data Fig. 7 [file 44319_2025_546_MOESM10_ESM.zip › Figure 7/Fig7F-Western GAPDH.png]

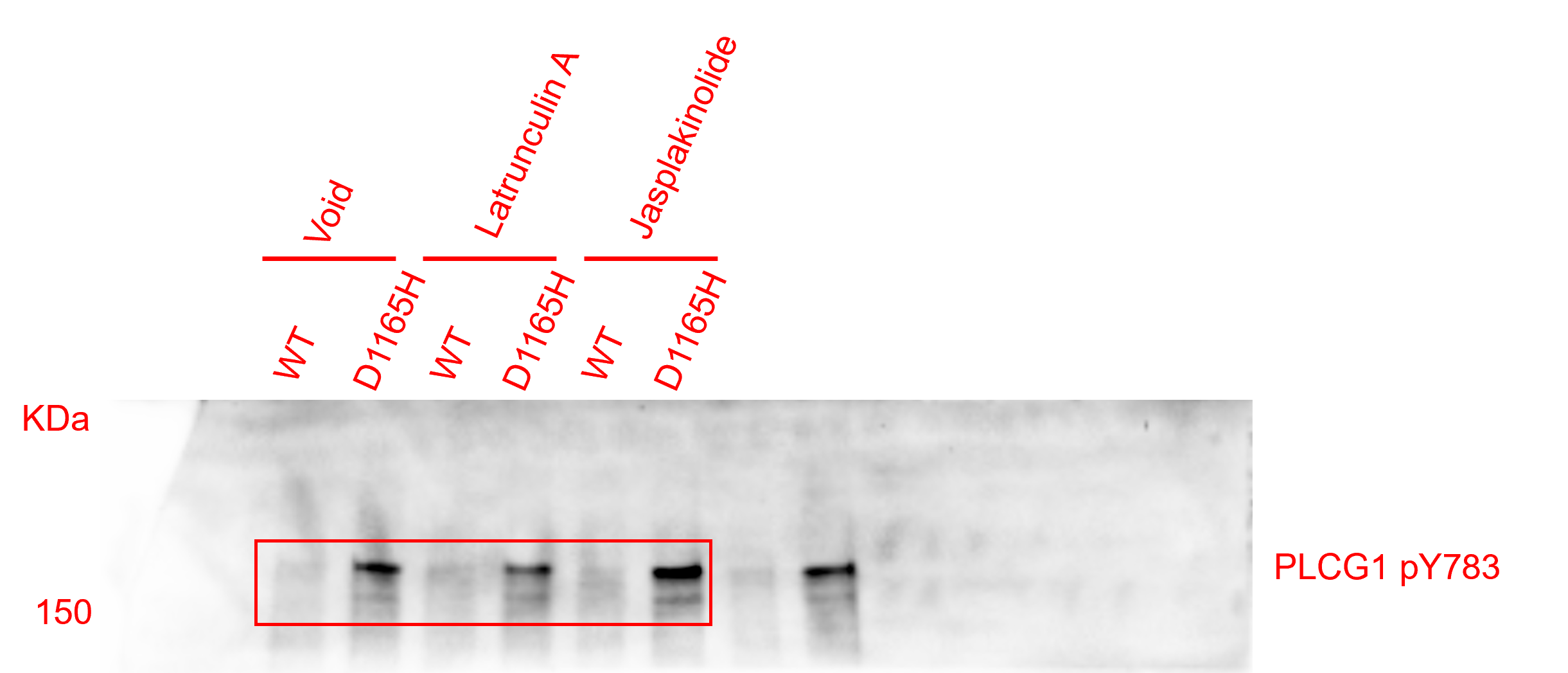

Supplement: Supplementary file 10 — Source data Fig. 7 [file 44319_2025_546_MOESM10_ESM.zip › Figure 7/Fig7F-Western PLCG1 pY783.png]

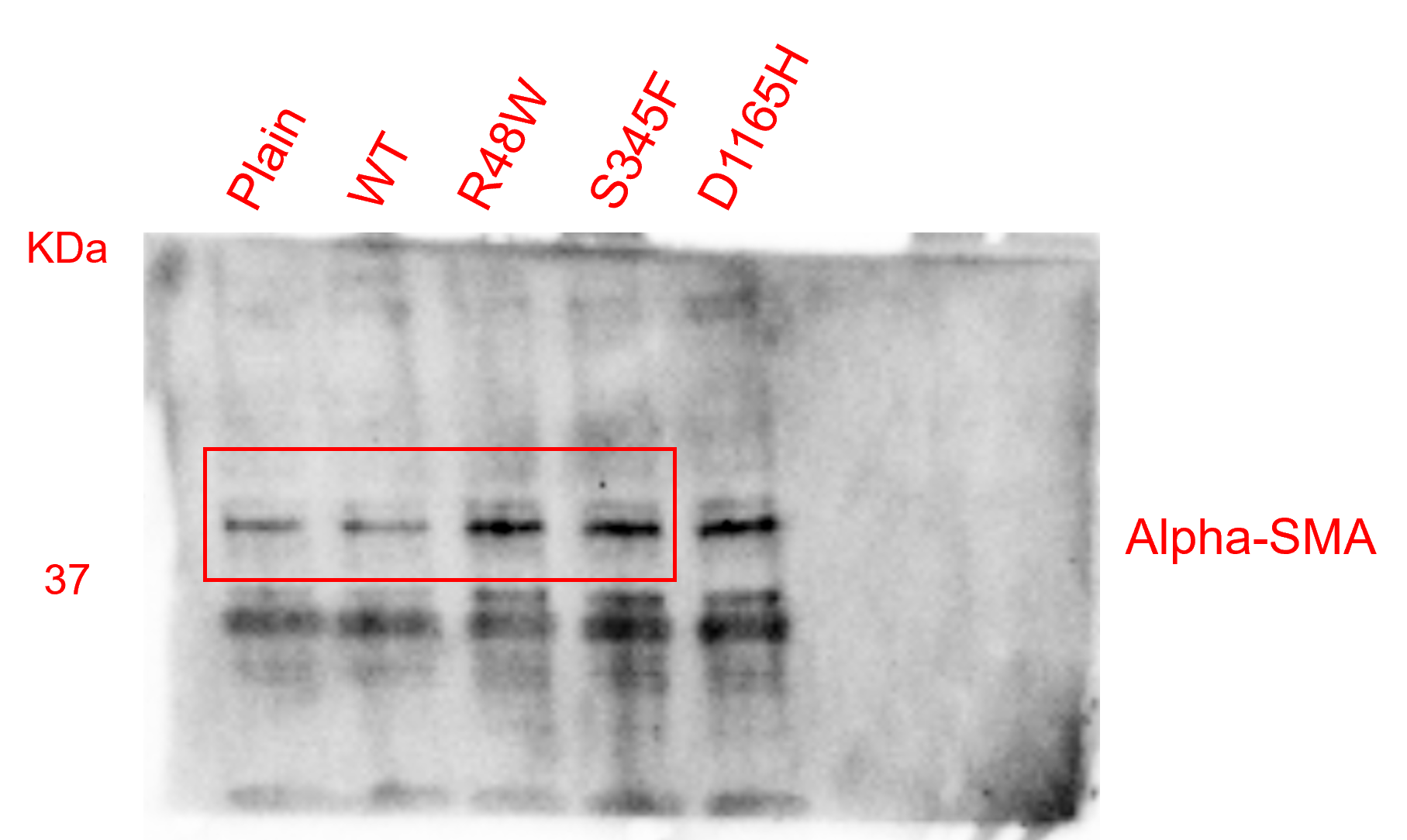

Supplement: Supplementary file 11 — Source data Fig. 8 [file 44319_2025_546_MOESM11_ESM.zip › Figure 8/Fig8A-Western Alpha-SMA.png]

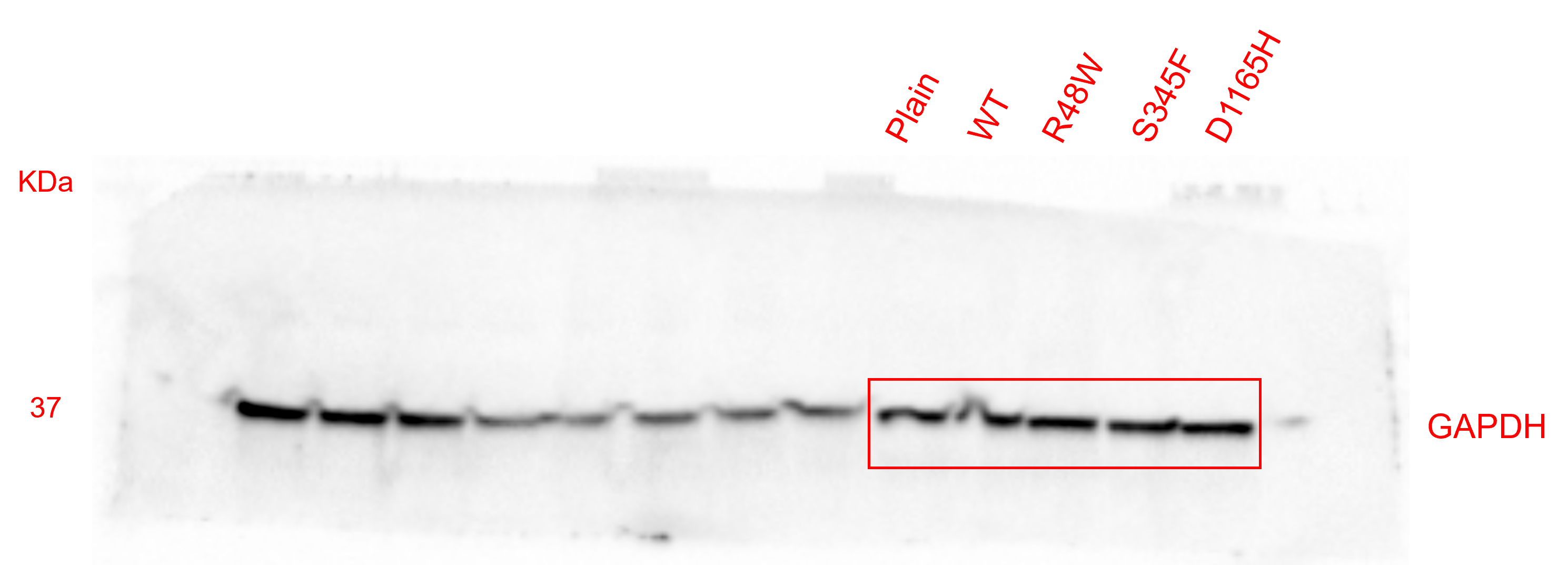

Supplement: Supplementary file 11 — Source data Fig. 8 [file 44319_2025_546_MOESM11_ESM.zip › Figure 8/Fig8A-Western GAPDH for Alpha-SMA.png]

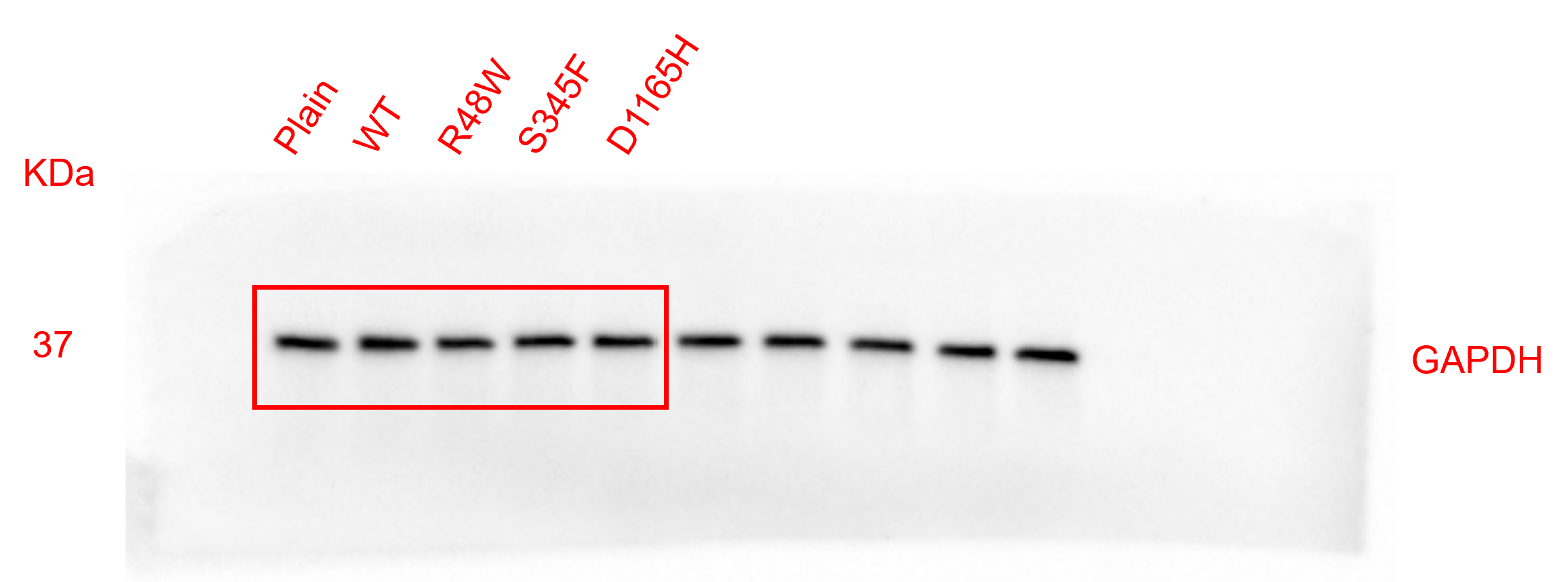

Supplement: Supplementary file 11 — Source data Fig. 8 [file 44319_2025_546_MOESM11_ESM.zip › Figure 8/Fig8A-Western GAPDH.png]

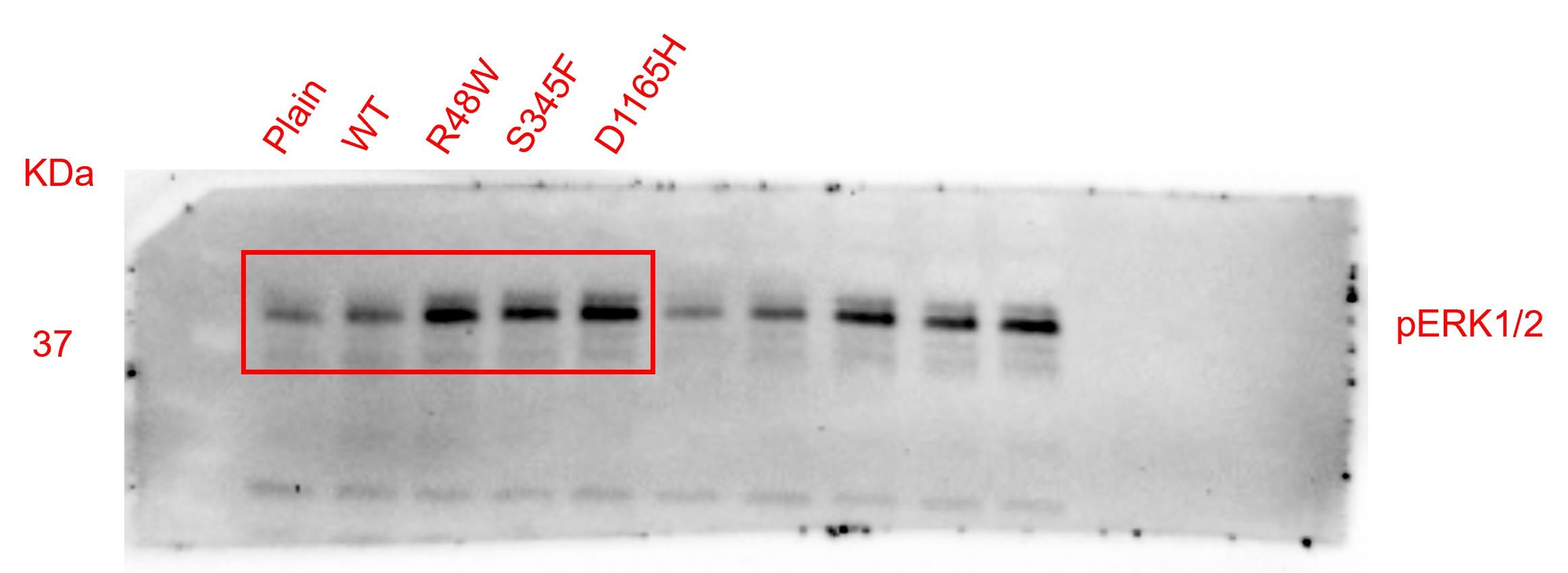

Supplement: Supplementary file 11 — Source data Fig. 8 [file 44319_2025_546_MOESM11_ESM.zip › Figure 8/Fig8A-Western pERK.png]

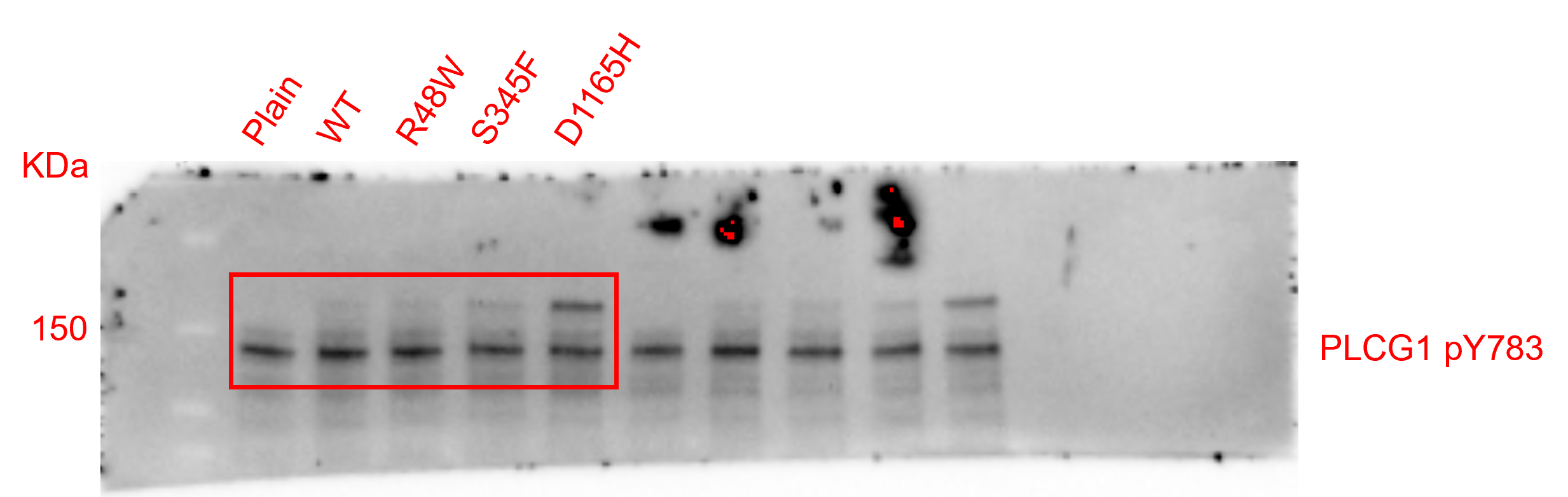

Supplement: Supplementary file 11 — Source data Fig. 8 [file 44319_2025_546_MOESM11_ESM.zip › Figure 8/Fig8A-Western PLCG1 pY783.png]

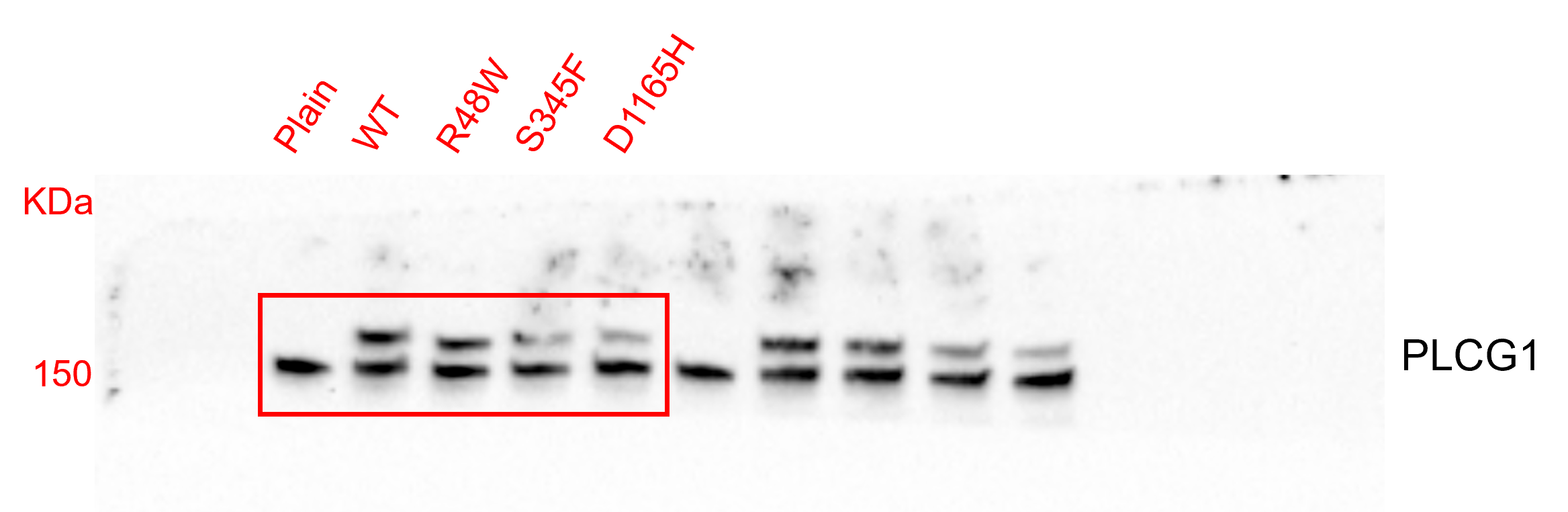

Supplement: Supplementary file 11 — Source data Fig. 8 [file 44319_2025_546_MOESM11_ESM.zip › Figure 8/Fig8A-Western PLCG1.png]
