# Supplementary material for: Hyperactive PLCG1 induces cell-autonomous and bystander T cell activation and drug resistance
Source: EMBO Rep. 2025 Aug 12;26(18):4563–86. doi: 10.1038/s44319-025-00546-x (PMC12457681; doi:10.1038/s44319-025-00546-x)
Supplement: Supplementary file 12 — Expanded View Figures [file 44319_2025_546_MOESM12_ESM.pdf]

Expanded View Figures

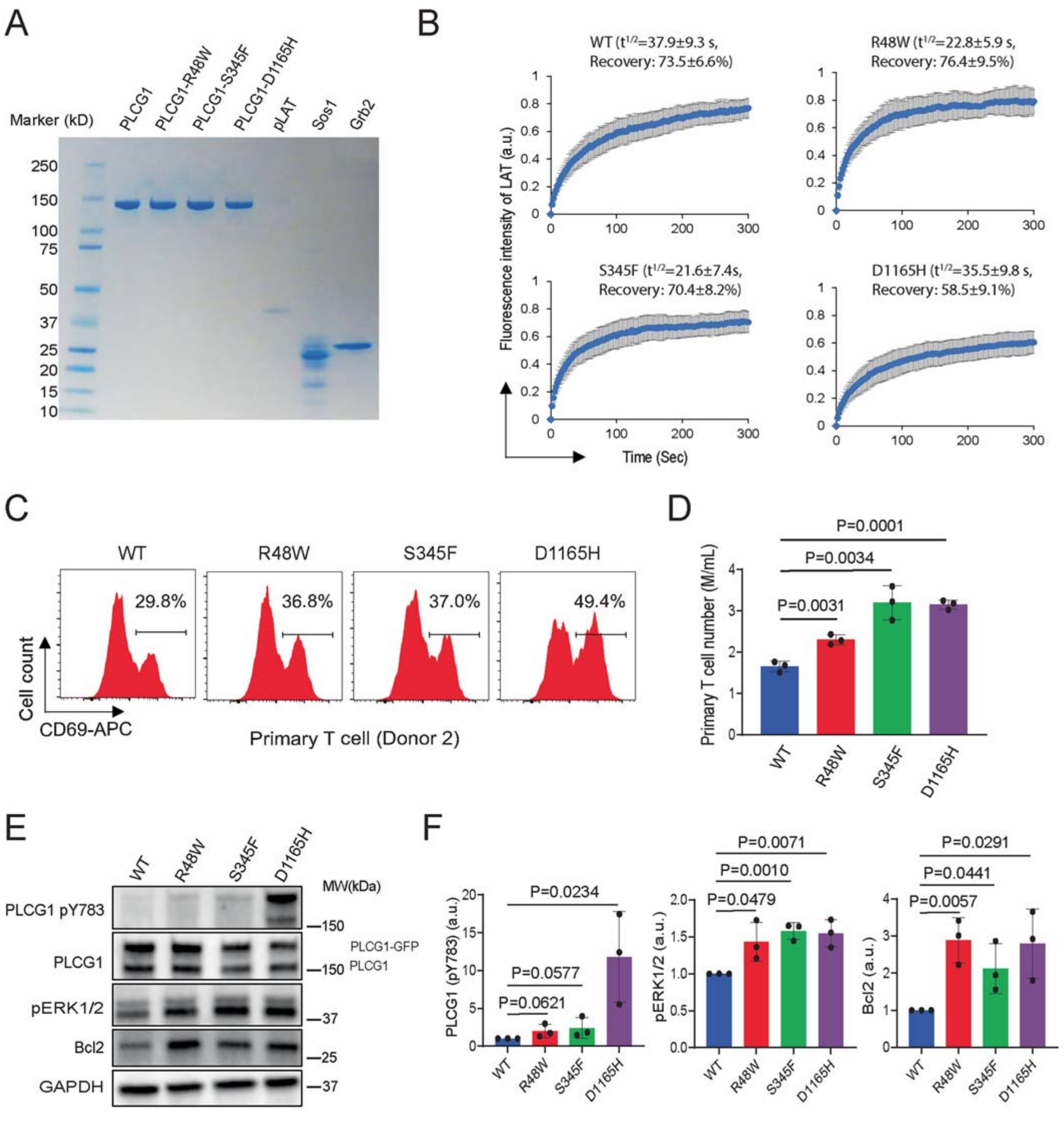

Figure EV1. PLCG1 mutants promote the activation of T cells.

(**A**) Recombinant proteins used in this study. The purified proteins were loaded to SDS-PAGE followed by Coomassie Blue staining. (**B**) FRAP analysis of LAT condensates. Shown are mean ± SD from $n = 10$ condensates. (**C**) Activation of human primary T cells expressing PLCG1 WT or mutants. The expression of CD69 was determined by flow cytometry 14 days after T cells were infected with lentivirus encoding PLCG1 WT or mutants. This is a repeated experiment using T cells from a different donor than what was used in Fig. 2G. (**D**) Proliferation of human primary T cells expressing PLCG1 WT or mutants. The cell number was quantified 14 days after T cells were infected with lentivirus encoding PLCG1 WT or mutants. This is a repeated experiment using T cells from a different donor than what was used in Fig. 2H. Shown are mean ± SD from $n = 3$ biological replicates. Unpaired two-tailed t test was used. (**E**) Immunoblot analysis of signaling in Hut78 cells ectopically expressing GFP-tagged PLCG1 WT or mutants (without TCR activation). Low-titer virus was used so that PLCG1 WT or mutants were expressed at a similar level to the endogenous PLCG1. (**F**) Quantification of (**E**). Shown are mean ± SD from $n = 3$ biological replicates. Unpaired two-tailed t-test was used.

© The Author(s)

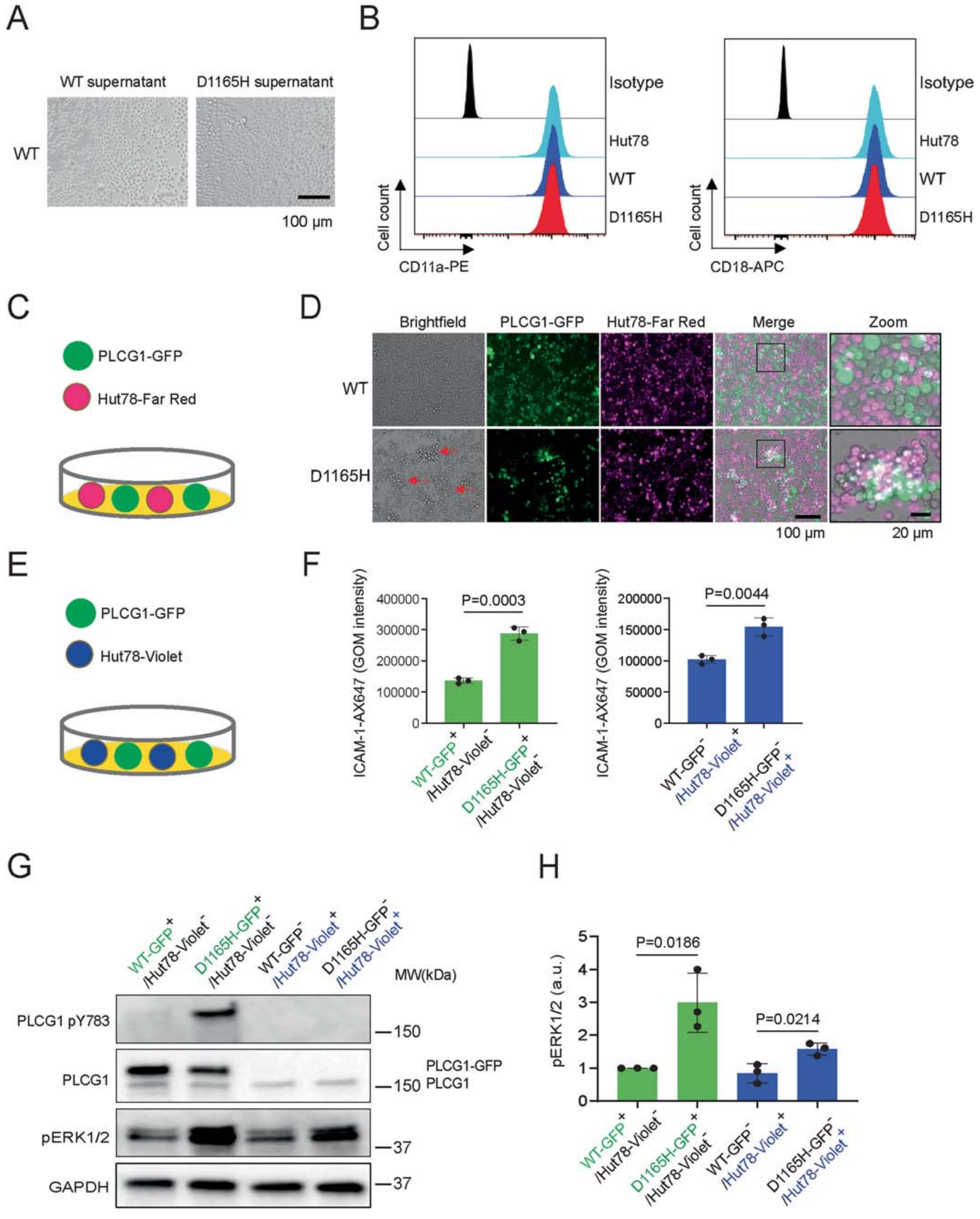
A
WT supernatant
D1165H supernatant
WT
100 µm
B
Isotype
Hut78
WT
D1165H
Cell count
CD11a-PE
CD18-APC
C
PLCG1-GFP
Hut78-Far Red
D
Brightfield
PLCG1-GFP
Hut78-Far Red
Merge
Zoom
WT
D1165H
100 µm
20 µm
E
PLCG1-GFP
Hut78-Violet
F
ICAM-1-AX647 (GOM intensity)
P=0.0003
P=0.0044
WT-GFP+/Hut78-Violet−
D1165H-GFP+/Hut78-Violet−
WT-GFP−/Hut78-Violet+
D1165H-GFP−/Hut78-Violet+
G
MW(kDa)
PLCG1 pY783
PLCG1
pERK1/2
GAPDH
PLCG1-GFP
150
37
H
pERK1/2 (a.u.)
P=0.0186
P=0.0214

Figure EV2. Hut78 cells expressing PLCG1 mutations induce aggregation and activation of neighboring cells expressing the wild-type PLCG1.

(**A**) Conditioned media from Hut78 cells expressing D1165H did not induced cell aggregation. Hut78 cells expressing PLCG1 WT were cultured in supernatant from Hut78 cells expressing the WT or D1165H PLCG1 for 2 days. (**B**) The expression of LFA-1 (Two subunits, CD11a and CD18) on the cell surface of Hut78 was detected by flow cytometry. (**C**) Schematics of co-culture assay. Hut78 cells harboring PLCG1 WT or D1165H were co-cultured with Far-red dye-labeled plain Hut78 cells at 1:1 ratio. (**D**) Plain Hut78 cells co-aggregated with Hut78 expressing D1165H. Red arrow indicates larger cell aggregate. Scale bar: 100 µm. An enlarged inset is shown on the right. Scale bar: 20 µm. (**E**) Schematics of the co-culture assay. Violet labeled Hut78 cells were co-cultured with Hut78 cells expressing PLCG1-GFP WT or D1165H in a 1:1 ratio for 72 h. (**F**) ICAM-1 cell surface expression by FACS. Shown are mean ± SD from $n = 3$ biological replicates. Unpaired two-tailed t-test was used. (**G**) The co-culture cells were sorted by FACS, lysed, and analyzed by Western blot. (**H**) Quantification of ERK phosphorylation from (**G**). Shown are mean ± SD from $n = 3$ biological replicates. Unpaired two-tailed t-test was used.

 © The Author(s)

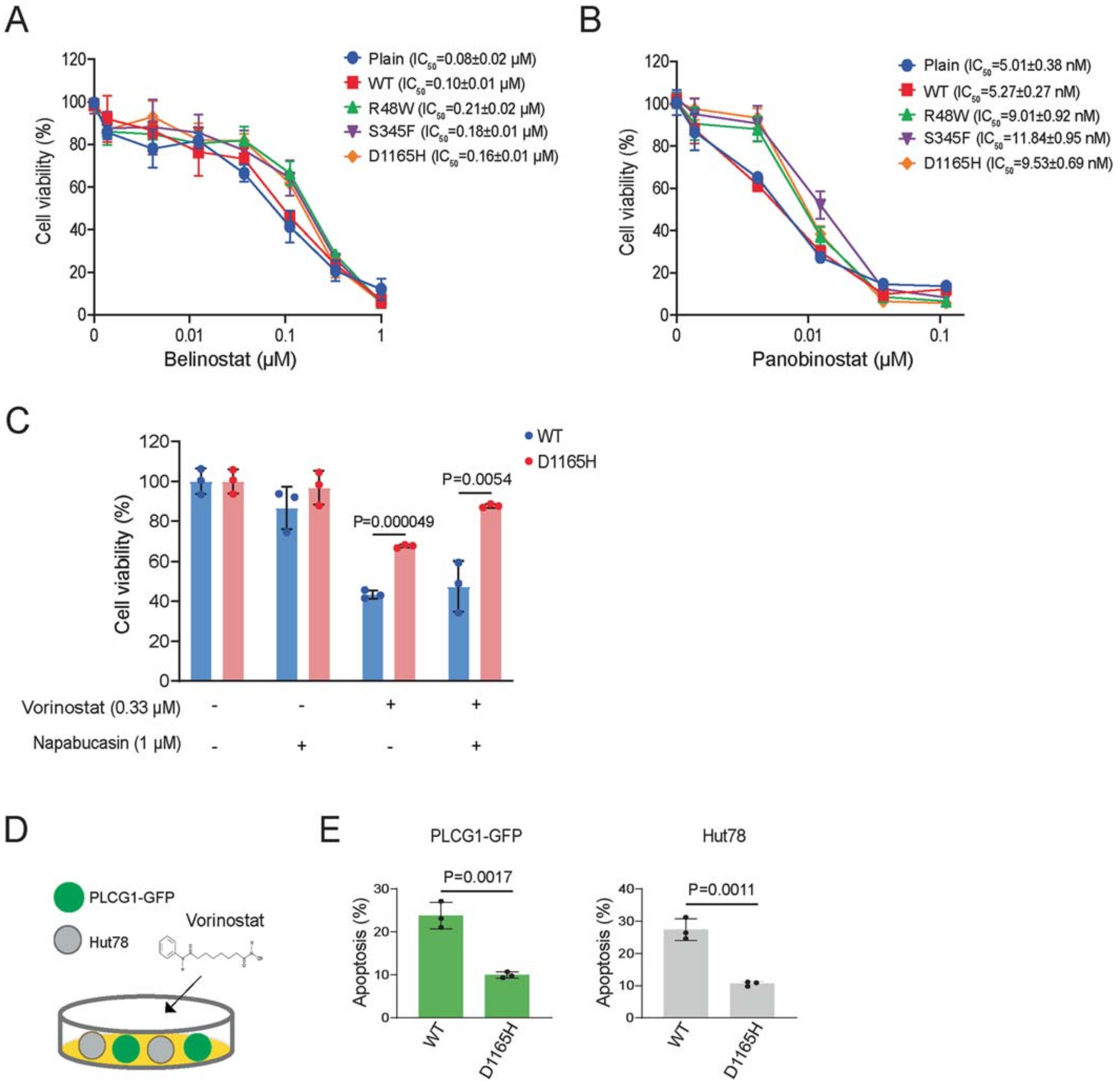

Figure EV3. PLCG1 mutations confer Hut78 resistance to HDAC inhibitors.

(**A**) PLCG1 mutations conferred Hut78 resistance to belinostat. The plain group is Hut78 cells without ectopically expressed PLCG1. The CCK8 assay was used to detect viable cell number after belinostat treatment for 72 h. Shown are mean ± SD from $n = 3$ biological replicates. (**B**) PLCG1 mutations conferred Hut78 resistance to panobionostat. The plain group is Hut78 cells without ectopically expressed PLCG1. The CCK8 assay was used to detect viable cell number after panobinostat treatment for 72 h. Shown are mean ± SD from $n = 3$ biological replicates. (**C**) STAT3 inhibitor napabucasin did not affect resistance to vorinostat. Shown are mean ± SD from $n = 3$ biological replicates. Unpaired two-tailed t-test was used. (**D**) Schematics of the co-culture assay with vorinostat treatment. Plain Hut78 cells (grey) were co-cultured with Hut78 cells expressing PLCG1-GFP WT or D1165H in a 1:1 ratio for 1 day, and then treated with 1 μM vorinostat for 48 h before being analyzed for apoptosis marker. (**E**) Hut78 cells expressing PLCG1 D1165H protected the neighboring plain Hut78 cells from vorinostat-induced apoptosis. Hut78 cells expressing PLCG1 WT or D1165H were co-cultured with plain Hut78 cells at 1:1 ratio. The apoptosis level, as indicated by annexin V staining, was determined by flow cytometry. Shown are mean ± SD from $n = 3$ biological replicates. Unpaired two-tailed t-test was used.

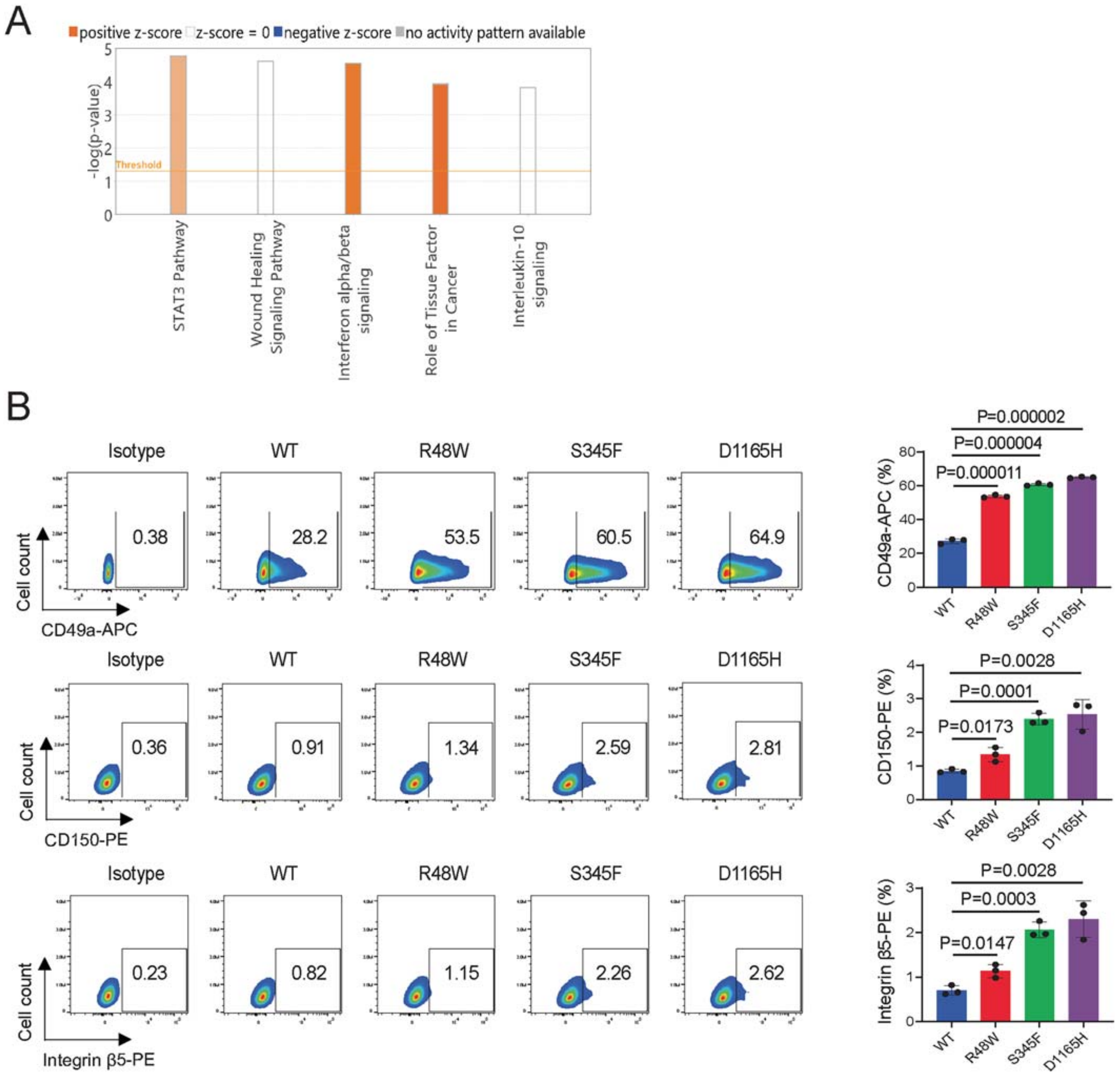

Figure EV4. Overlapped genes between hyperactive PLCG1 signaling and TCR signaling.

(**A**) Qiagen ingenuity pathway analysis (IPA) showed pathways enriched in overlapping genes between hyperactive PLCG1 signaling and TCR signaling. (**B**) Cell surface protein expression by flow cytometry. Shown are mean ± SD from $n = 3$ biological replicates. Unpaired two-tailed t test was used.

 © The Author(s)

A

Cell line	Gene variant	Codon Change	Mutation type	Amino acid mutation	Allele frequency-gnomAD database	dbSNP ID
Jurkat	PLCG1-T2438C	aTc/aCc	T(0%)/C(100%), Homozygous	PLCG1-I813T	58.3%, Common variant (>5%)	rs753381
Hut78	PLCG1-A835G	Agc/Ggc	A(43%)/G(57%), Heterozygous	PLCG1-S279G	15.4%, Common variant (>5%)	rs2228246
Hut78	PLCG1-T2438C	aTc/aCc	T(43%)/C(57%), Heterozygous	PLCG1-I813T	58.3%, Common variant (>5%)	rs753381

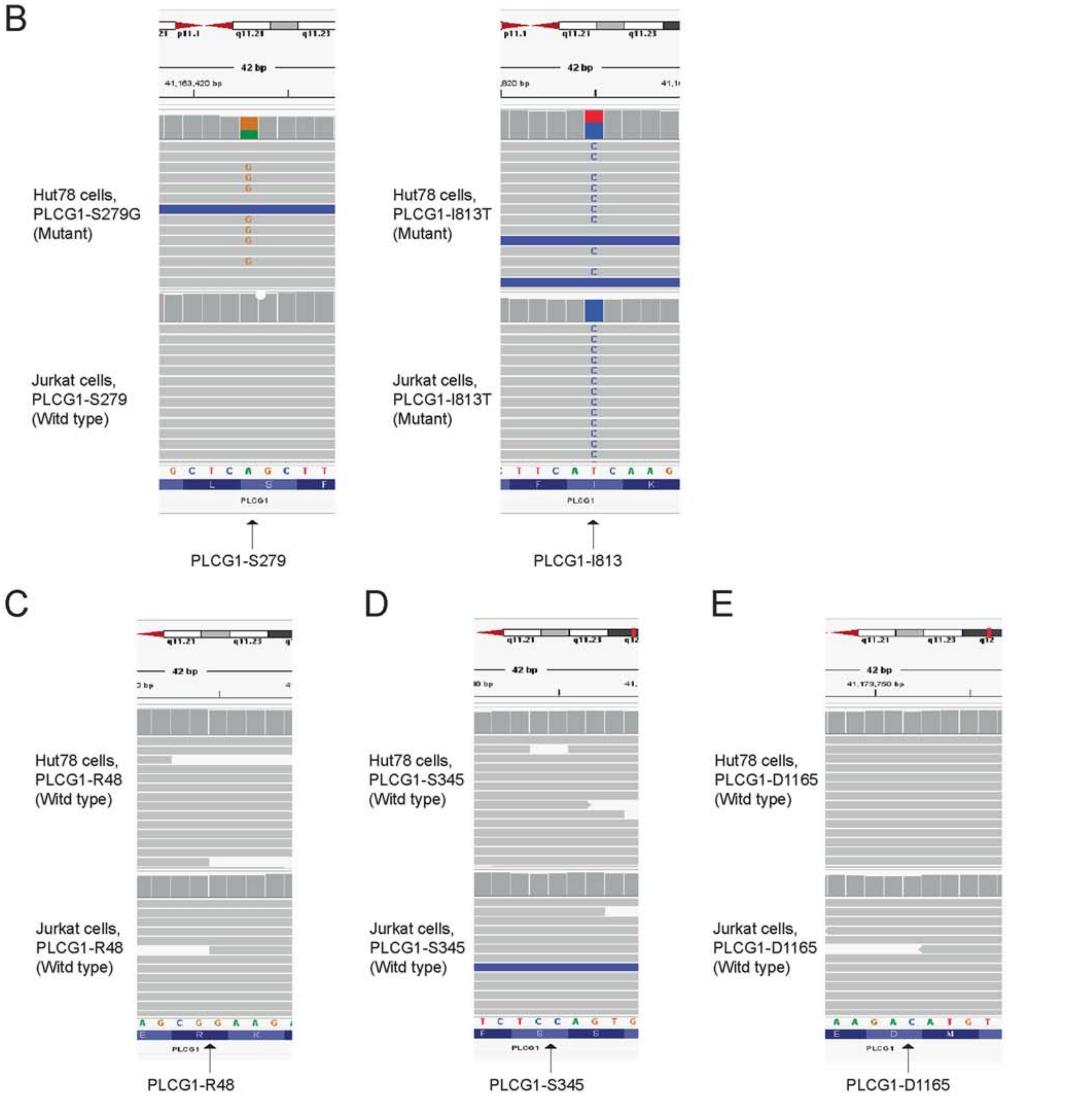

Figure EV5. PLCG1 sequence in Jurkat and Hut78 cells as revealed by whole-genome sequencing.

(**A**) Common variants of PLCG1 in Jurkat and Hut78 cells. (**B**) Local view of the PLCG1 common variants in Jurkat and Hut78 cells as compared to reference human genome sequence. (**C**) Local view of sequences encoding PLCG1-R48. (**D**) Local view of sequences encoding PLCG1-S345. (**E**) Local view of sequences encoding PLCG1-D1165.

 © The Author(s)
